# Molecular Interactions Stabilizing the Promatrix Metalloprotease-9·Serglycin Heteromer

**DOI:** 10.3390/ijms21124205

**Published:** 2020-06-12

**Authors:** Rangita Dawadi, Nabin Malla, Beate Hegge, Imin Wushur, Eli Berg, Gunbjørg Svineng, Ingebrigt Sylte, Jan-Olof Winberg

**Affiliations:** Department of Medical Biology, Faculty of Health Sciences, UiT-The Arctic University of Norway, 9037 Tromsø, Norway; rangita.dawadi@uit.no (R.D.); nabin.malla@uit.no (N.M.); beate.hegge@uit.no (B.H.); imin.wushur@uit.no (I.W.); elibrg@online.no (E.B.); gunbjorg.svineng@uit.no (G.S.); ingebrigt.sylte@uit.no (I.S.)

**Keywords:** serglycin, proMMP-9, proMMP-9 deletion variants, proMMP-9 complexes, in vitro reconstitution, peptide arrays, molecular modelling, docking, molecular dynamics simulation

## Abstract

Previous studies have shown that THP-1 cells produced an SDS-stable and reduction-sensitive complex between proMMP-9 and a chondroitin sulfate proteoglycan (CSPG) core protein. The complex could be reconstituted in vitro using purified serglycin (SG) and proMMP-9 and contained no inter-disulfide bridges. It was suggested that the complex involved both the FnII module and HPX domain of proMMP-9. The aims of the present study were to resolve the interacting regions of the molecules that form the complex and the types of interactions involved. In order to study this, we expressed and purified full-length and deletion variants of proMMP-9, purified CSPG and SG, and performed in vitro reconstitution assays, peptide arrays, protein modelling, docking, and molecular dynamics (MD) simulations. ProMMP-9 variants lacking both the FnII module and the HPX domain did not form the proMMP-9∙CSPG/SG complex. Deletion variants containing at least the FnII module or the HPX domain formed the proMMP-9∙CSPG/SG complex, as did the SG core protein without CS chains. The interacting parts covered large surface areas of both molecules and implicated dynamic and complementary ionic, hydrophobic, and hydrogen bond interactions. Hence, no short single interacting linear motifs in the two macromolecules could explain the strong SDS-stable and reduction-sensitive binding.

## 1. Introduction

The matrix metalloprotease (MMP) or matrixin family is a group of extracellular zinc and calcium dependent metallo-enzymes expressed by most cells and tissues. In humans, there are 23 different MMPs, seven of which are membrane bound and 16 forms that are secreted, with MMP-9 being one of the secreted variants [1]. The MMPs as a group, as well as individual enzymes, have broad substrate specificity, and together, they are able to degrade almost all extracellular matrix proteins. In addition, they cleave a number of non-extracellular matrix molecules, such as cytokines, chemokines, adhesion molecules, cell receptors, proteases (including MMPs), protease inhibitors, and a number of intracellular proteins [1,2,3,4,5,6,7,8]. Due to their broad substrate specificity, MMPs play a critical role in cell and tissue homeostasis and normal development, while dysregulation during disease may be either detrimental or protective to the organism. Their dual role in diseases appears to depend on various factors such as their physical location, the timeframe of their activity, and the substrate cleaved [1,4,5,6,7,9,10,11,12,13,14,15,16,17,18]. The numerous interactions between proteins and other molecules regulate their accessibility to proteolytic degradation, by either hiding protease cleavage sites or exposing new sites. Similarly, proteases form interactions with molecules in the extracellular matrix. This may affect their location, substrate specificity, and catalytic efficiency [1,19,20,21,22,23,24,25,26,27].

MMP-9 is secreted as an inactive proenzyme from various types of cells and, like the other MMPs, contains an N-terminal prodomain, a catalytic domain, and a C-terminal hemopexin-like (HPX) domain. In addition, MMP-9 contains a module in the catalytic domain of three fibronectin-II-like repeats (FnII) and a unique highly O-glycosylated hinge domain (OG) that connects the catalytic and the HPX domains [1,28]. The OG domain is very flexible as shown by small-angle X-ray scattering (SAXS) and atomic force microscopy (AFM) [29] and is the likely reason that a 3D structure of the entire protein is lacking. However, the 3D structures of the MMP-9 HPX domain [30] and of a mini-MMP-9 consisting of the pro- and catalytic domain including the FnII module are known [31].

ProMMP-9 is secreted from cells as a monomer and an SDS-stable (reduction-sensitive) homodimer/homotrimer [28,32,33,34]. In addition, proMMP-9 is known to form heteromer complexes with various types of molecules such as TIMP-1 and TIMP-3 (tissue inhibitors of metalloproteases), lipocalin/NGAL (neutrophil gelatinase associated lipocalin), haptoglobin, proMMP-1, proMMP-8, heparin, and various proteoglycan (PG) core proteins [19,23]. The human leukemic monocyte cell-line (THP-1) secretes proMMP-9 as a monomer, homodimer/homotrimer, and as heteromers with one or several chondroitin sulfate PG (CSPG) core proteins [35,36]. The homodimer/homotrimer and the heteromers are SDS-stable and reduction-sensitive. Previously, it was shown that calcium, which is known to stabilize MMP-9, induced activation of proMMP-9 bound to the CSPG, but not of unbound proMMP-9 [37]. The Ca^2+^-induced activation of proMMP-9 bound to the CSPG core protein resulted in removal of the prodomain, followed by a stepwise truncation of the HPX domain. The size of the MMP-9 fragments also suggested that parts of the CSPG core protein were cleaved, but still remained bound to the truncated MMP-9. Furthermore, APMA, which is an activator of proMMP-9, could not activate proMMP-9 bound to the CSPG, but instead prevented the Ca^2+^-induced activation [37]. This indicated that the prodomain might be involved in the complex formation along with the HPX domain. It was also shown that the CSPG core protein of the formed proMMP-9∙CSPG complex hides the region of the FnII module involved in gelatin binding [38]. This suggests that the CSPG also binds to the enzyme through the FnII module.

ProMMP-9∙CSPG complexes can be reconstituted *in vitro* by mixing proMMP-9 purified from THP-1 cells with isolated CSPGs from the leukemic monocyte cell lines THP-1, U-937, and MonoMac, as well as the two purified CSPGs, serglycin (SG) from human myeloma cells, and versican from normal human aortas [39]. The *in vitro* reconstitution resulted in two types of proMMP-9∙CSPG complexes, one SDS-stable and reduction-sensitive, and the other SDS-soluble. The *in vitro* reconstitution of the complexes showed that the reduction-sensitive complexes were not due to the formation of a disulfide bridge between the two proteins, but rather due to a combination of ionic and hydrophobic interactions. Gelatin inhibited the formation of both types of complexes, while TIMP-1 only inhibited the formation of the SDS-soluble complex. This suggests that both the FnII module and the HPX domain are involved in the complex formation.

Various cell types, such as hematopoietic and endothelial cells, produce the proteoglycan SG. At physiological conditions, SG has a role in the immune system, in hemostasis, cell growth, apoptosis, and reproduction, as well as in diseases such as cancer, inflammatory disorders, as well as platelet-associated disorders [21,40,41,42,43,44]. The glycosaminoglycan (GAG) chains associated with the core protein are either chondroitin sulfate (CS), heparin/heparan sulfate (HS), or a mixture of the two depending on the cell type [21]. In hematopoietic cells such as the leukocytic monocyte cell line THP-1, the GAG chains associated with the proteoglycan core protein are CS [21,38]. The main CSPG produced by THP-1 monocytes is SG, and this contributes to more than 95% of the secreted CSPGs [45,46]. In human and mouse cells, the SG core protein is transcribed from three exons where exon 1 codes for the signal peptide (amino acids 1–27), which is removed in the endoplasmatic reticulum (ER) during secretion [21]. In humans, exon 2 codes for amino acids 28–76 and exon 3 for amino acids 77–158, and the eight Ser-Gly repeats are from amino acids 94–111 [21]. In THP-1 cells, SG is secreted with a small core protein that contains 131 amino acids. The molecular mass of this core protein is approximately 14 kDa. Therefore, in this paper, we numbered the SG amino acid sequence from 1–131 and the eight Ser-Gly repeats 67–84. The GAG chains are attached to serine residues, which are clustered as eight Ser-Gly repeats in the center of the core protein [21,47]. Both SG and MMP-9 are inflammatory proteins. To some extent, they are produced in the same tissues and by the same cells.

**Figure 1:**
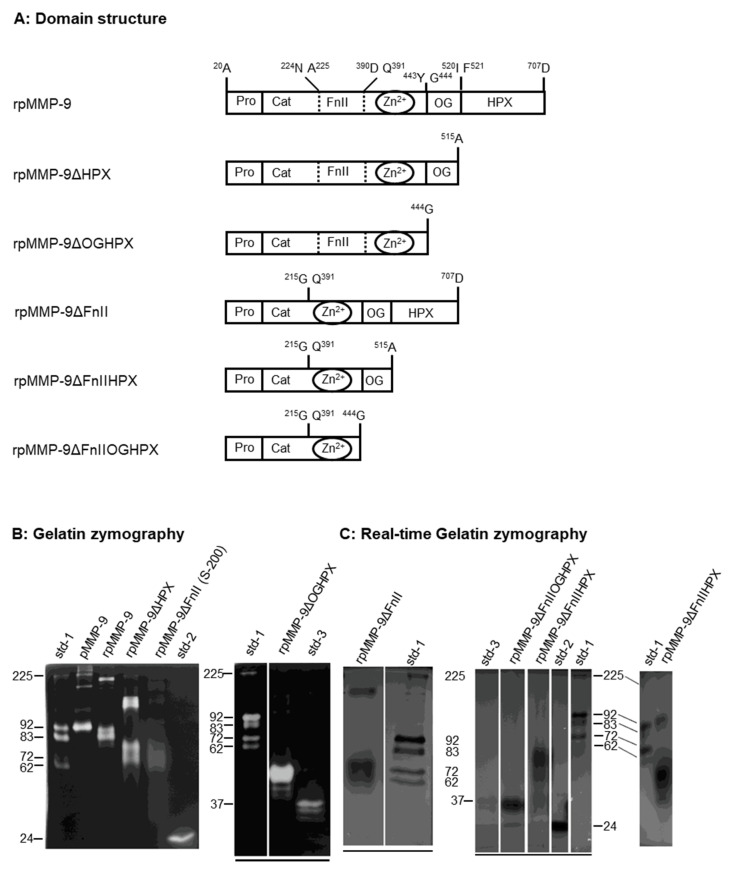
Schematic domain structure (**A**), gelatin zymography (**B**), and real-time gelatin zymography (**C**) of recombinant proMMP-9 variants. (**A**) At the top, the full-length proMMP-9 with its domains. Shown above in (**A**) is the numbering of the amino acids starting with the pre domain, which is cleaved off in the endoplasmatic reticulum, and the mature proenzyme, which starts at amino acid 20 and ends at amino acid 707. Shown also are the amino acids at the border of the FnII module and at the boarder of the OG and the HPX domain based on Vandooren et al. 2013 [28]. The five deletion variants with their C-terminal amino acid are shown. In the fibronectin-deleted variants, the amino acids that were linked together after the FnII deletion are shown. The different recombinant proMMP-9 (rpMMP-9) variants were produced in Sf9 and High Five insect cells with a baculoviral transfection system as described in the Materials and Methods. (**B**) The purified variants were applied to gelatin zymography, and the rpMMP-9ΔFnII variant was from the pooled fractions from a Sepharose S-200 column. Under these conditions, this variant contained mainly the monomeric variant. (**C**) Real-time gelatin zymography of crude media of the three ΔFnII-deleted variants. Standards in (**B**,**C**) are: std-1 (a mixture of MMP-9 (homodimer/homotrimer 225 kDa; monomeric pro 92 kDa; and active 83 kDa) from THP-1 cells and MMP-2 (pro 72 kDa; active 62 kDa) from skin fibroblasts; std-2 (trypsin 24 kDa); and std-3 (catalytic domain of MMP-9 containing the FnII repeats 37 kDa). A line under gels indicates that the samples are from the same gel, which was cut and pasted for clarity in photoshop.

The main aim of the present study was to resolve the molecular interactions between proMMP-9 and SG in the proMMP-9∙CSPG complex. This knowledge is important for the understanding of why the two macromolecules form a strong complex. In general, such information may be used to generate inhibitors acting at MMP-9 substrate exosites instead of the catalytic site. To solve the aims in the present work, we purified proMMP-9 from THP-1 cells and produced and purified recombinant full-length proMMP-9 and five recombinant deletion variants. The deletion variants lack either the C-terminal HPX domain, the HPX, and the hinge region (OG domain) or the FnII-like module. In addition, one variant lacks both the FnII module and the HPX domain, while another variant lacks the FnII module in addition to the OG and HPX domains (Figure 1). These variants were used for *in vitro* reconstitution experiments with isolated CSPGs and purified SG to study which regions of the enzyme were involved in the complex formation. Special focus was on the FnII module and the HPX domain in order to determine if both domains/modules were necessary for establishing the SDS-stable and SDS-soluble complexes or if only one of these domains/modules was necessary. *In vitro* reconstitution experiments with recombinant His-tagged SG (Ht-SG) lacking GAG chains were also performed in order to determine if CS chains were necessary for the complex formation. Peptide arrays, protein modelling, docking, and molecular dynamics (MD) simulation experiments were performed to determine which parts of the SG core protein, the FnII module, and the HPX domain of proMMP-9 were involved in the complex formation. These experiments revealed that a proMMP-9∙CSPG/SG complex was not formed if both the FnII module and the HPX domain in proMMP-9 were absent. Furthermore, no short single interacting linear motifs in the two macromolecules could explain the strong SDS-stable and reduction-sensitive binding that hold together the proMMP-9∙CSPG/SG complex.

## 2. Results and Discussion

### 2.1. Expression and Purification of Recombinant Domain Variants of Human proMMP-9 and Full-Length proMMP-9 from THP-1 Cells

**Figure 2:**
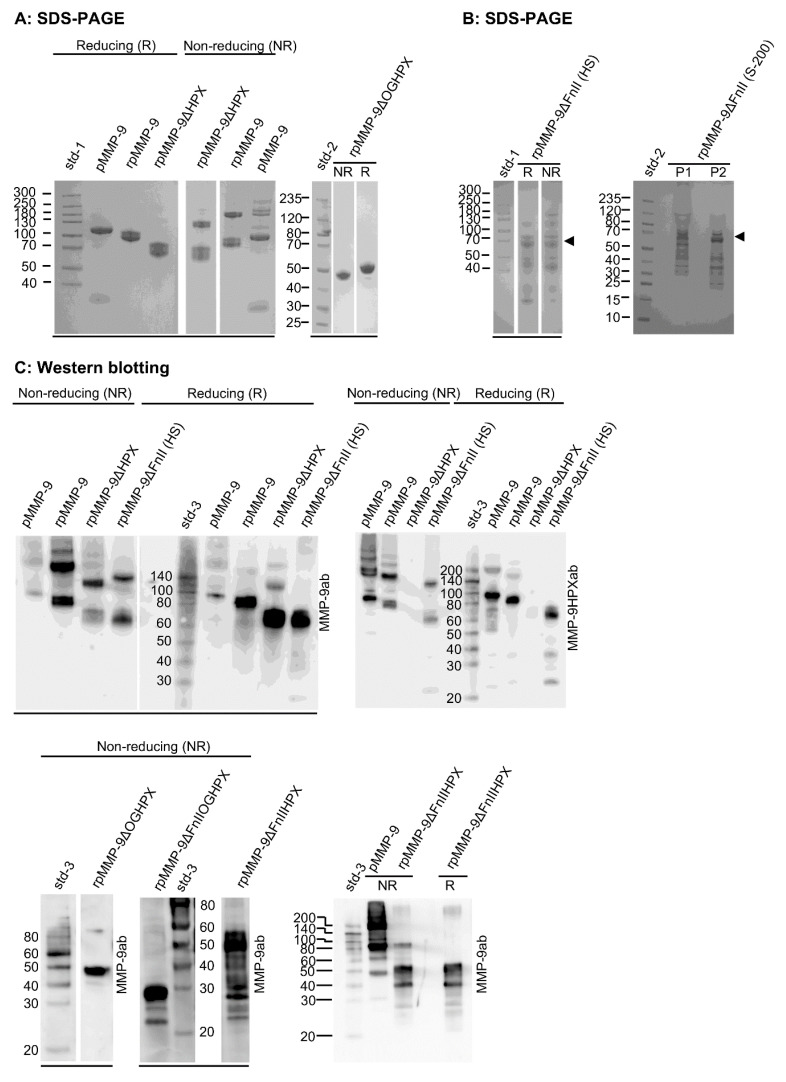
SDS-PAGE and Western blot analysis of recombinant proMMP-9 variants. After purification, the different recombinant proMMP-9 (rpMMP-9) variants shown in Figure 1 were analyzed by Imperial stained SDS-PAGE (**A**,**B**) and Western blotting (**C**). To determine the purity, the molecular mass, as well as the presence of monomers and homomultimers, reducing (R) and non-reducing (NR) conditions were used. As a control, purified pMMP-9 (proMMP-9 from THP-1 cells) was used, which also contained small amounts of TIMP-1 (30 kDa), as seen in (**A**). Std-1 and Std-2 are the high range and broad range molecular weight standards from Thermo Scientific, respectively, with the molecular weights in kDa shown (**A**,**B**), and Std-3 in (**C**) is the biotinylated protein ladder. In (**C**), MMP-9ab and MMP-9HPXAb are polyclonal antibodies against the entire enzyme and the C-terminal HPX domain, respectively. In (**B**,**C**), recombinant proMMP-9ΔFnII is purified on either a Sephacryl-S-200 column (rpMMP-9ΔFnII (S-200)) or a heparin-Sepharose column (rpMMP-9ΔFnII (HS)), as described in the Materials and Methods. In (**C**), the crude media of two FnII-deletion variants (rpMMP-9ΔFnIIHPX and rpMMP-9ΔFnIIOGHPX) are used. In (**B**), the arrowhead shows the position of the rpMMP-9ΔFnII monomer. P1 and P2 are pooled non-reduced fractions from the S-200 column, and both contained rpMMP-9ΔFnII based on gelatin zymography. The line under gels indicates that the samples are from the same gel, which was cut and pasted for the sake of clarity in Photoshop.

Production and purification of recombinant full-length proMMP-9 (rpMMP-9) were performed as described previously [48]. The recombinant deletion variants of proMMP-9 (Figure 1A) were generated and purified as described in the Materials and Methods. Production and purification of proMMP-9 from THP-1 cells (pMMP-9) were performed as described previously [38,48]. The purified and partly purified proMMP-9 variants were subjected to gelatin zymography (Figure 1B), SDS-PAGE, and Western blotting (Figure 2). In addition, crude media containing proMMP-9 deletion variants were subjected to real-time gelatin zymography (Figure 1C) and Western blotting (Figure 2).

The variants containing the FnII module in the catalytic domain were purified on a gelatin-Sepharose column. SDS-PAGE both under reducing and non-reducing conditions showed that the purified recombinant enzymes containing the FnII module (rpMMP-9, rpMMP-9ΔHPX, and rpMMP-9ΔOGHPX), as well as the proMMP-9 purified from THP-1 cells (pMMP-9) were almost homogeneous (Figure 2A). As we have shown in previous studies [38,48], the purified proMMP-9 from THP-1 cells (pMMP-9) contained a small amount of TIMP-1 (30 kDa). Gelatin was found to bind the FnII module in the catalytic domain [49,50,51,52,53], and it has also been suggested that gelatin binds the HPX domain in MMP-9 [54,55]. In these studies, recombinant murine and human HPX-9 domains were used [54,55]. Therefore, we first tried to purify the deletion variant that only lacked the FnII module (rpMMP-9ΔFnII) on a gelatin-Sepharose column. However, this variant did not bind the column under the conditions used, and therefore, several other purification methods were tried. A previous study reported that the full-length recombinant proMMP-9 isolated from Sf9 insect cells binds Helix pomatia agglutinin (HPA) in contrast to deletion variants of MMP-9 lacking the O-glycosylated hinge region (OG) [56]. In our hands, rpMMP-9ΔFnII did not bind the HPA-linked agarose beads. Therefore, two other methods were tested. In one method, the crude medium from the Sf9 cells containing proMMP-9ΔFnII was subjected to NH_4_SO_4_ precipitation followed by gel filtration using Sephacryl S-200 as described in the Materials and Methods. In the other method, the crude medium containing proMMP-9ΔFnII was first applied to a spin column with a 30 kDa cut-off and thereafter applied to a heparin-Sepharose column. None of these two methods resulted in a pure homogeneous product, as seen in Figure 2B. Therefore, in our further experiments with this deletion variant, either the partly purified enzyme (from the heparin-Sepharose column or the S-200 column) or the crude unpurified media were used. In all experiments with the two other deletion variants lacking the FnII module (rpMMP-9ΔFnIIHPX and rpMMP-9ΔFnIIOGHPX), crude unpurified media were used. As can be seen in Figure 1C and Figure 2C, the expressed triple deletion variant (rpMMP-9ΔFnIIOGHPX) had the expected molecular mass of around 28 kDa. The rpMMP-9ΔFnIIHPX with a theoretical molecular mass of the protein of around 35 kDa (without bound sugar chains) was shown in zymography with main activity bands around 50–60 kDa (Figure 1C) and in Western blot with two main MMP-9 bands, one with a molecular mass of 50 kDa and the other of around 55 kDa (Figure 2C). This suggested that glycosylation of the OG domain contributed with approximately 15–20 kDa to the molecular mass of the rpMMP-9ΔFnIIHPX enzyme. In addition, several minor bands with lower molecular size appeared (Figure 2C). The bands lower than 35 kDa indicated that the enzyme was partly processed during the expression. Notably, in some of the batches expressing the rpMMP-9ΔFnIIHPX deletion variant, in addition to the bands at 50–55 kDa, a band with a molecular mass around 100–120 kDa also appeared (Figure 1C and Figure 2C). Under reducing conditions, the band of around 100–120 kDa in the Western blot disappeared (Figure 2C), suggesting that it was a homodimer of the rpMMP-9ΔFnIIHPX protein.

Both SDS-PAGE (Figure 2A) and Western blotting (Figure 2C) under reducing and non-reducing conditions, as well as gelatin zymography (Figure 1B) and real-time gelatin zymography (Figure 1C) showed that recombinant variants containing the OG domain of proMMP-9 and the proMMP-9 from THP-1 cells formed a monomer and a reduction-sensitive homomultimer. The recombinant variants lacking the OG domain only formed monomers (Figure 1B,C, Figure 2A–C). This was consistent with a previous study of recombinant proMMP-9 deletion variants [56]. The size of the monomers of rpMMP-9ΔHPX and rpMMP-9ΔFnII deletion variants were almost identical, with a molecular mass around 70 kDa. The homomultimer of the rpMMP-9ΔFnII was slightly larger than the corresponding homomultimer of rpMMP-9ΔHPX. Previously, it was shown that the homomultimers were produced intracellularly and concomitantly with glycosylation [33]. The dimers/trimers could be separated from the monomers, and both forms were stable [33,34]. The reduction-sensitive homomultimer was assumed to be a homodimer linked by a disulfide bridge [32,33]. Recently, it was suggested that the reduction-sensitive proMMP-9 multimer with a molecular mass of approximately 225 kDa was not a dimer, but a disulfide linked cyclic homotrimer, although the presence of disulfide bridges was not directly proven [34]. The recombinant produced HPX domain of MMP-9 (HPX-9) also formed a monomer and a reduction-sensitive homodimer [30]. X-ray crystallography showed that the reduction-sensitive dimer was not linked by an inter-disulfide bridge, but by hydrophobic interactions and an ionic bond [30]. The reduction sensitivity occurred due to breaking of the intra-disulfide bridge between ^516^C and ^704^C. We did not try to identify the nature of the size difference between the homomultimers formed with rpMMP-9ΔHPX and rpMMP-9ΔFnII. As expected, the proMMP-9ΔHPX variant was not detected in Western blotting using an antibody against the MMP-9HPX domain (MMP-9HPXab), while the variants containing this domain were detected (Figure 2C). MS-MS analysis confirmed the amino acid sequences of the expressed protein variants. However, the ΔFnII variants were not subjected to MS analysis due to the presence of large impurities in these preparations that were partly purified.

### 2.2. Production and Purification of CSPG/SG and Serglycin

CSPG was produced by unstimulated THP-1 cells (a human leukemic monocyte cell line) [35], and the secreted CSPG was purified as described in the Materials and Methods. From the Q-Sepharose purified preparation of CSPG, SG was separated from other putative CSPGs using a Sephacryl S-400 column. The elution profile and the purity of the purified SG are shown in Figure S1. Fractions II-IV contained SG, but no versican, based on Western blotting analysis. In silver stained SDS-PAGE gels, a main band of approximately 26 kDa occurred in the chondroitin ABC lyase (cABC) treated material, corresponding to the size of the SG band(s) seen in the Western blots (Figure S1). Previously, we showed that the purified CSPG material from THP-1 cells in addition to SG also contained versican [39]. Versican is a large proteoglycan with a molecular mass ≥ 1000 kDa, consisting of a core protein with a molecular mass around 400 kDa and 12–15 CS chains attached along with N- and O-linked oligosaccharides [57]. As seen from Figure S1, if versican was present in the Q-Sepharose purified CSPG, it should be eluted in Faction I from the Sephacryl S-400 column. The amount of proteoglycans in the different fractions was determined by the Safranin O method. Based on this, the amount of proteoglycan in Fraction I was approximately 0.7% of the total amount of the produced proteoglycans. This suggested that the major secreted proteoglycan was SG, which fit well with previous studies showing that the main CSPG produced by THP-1 monocytes was SG [45,46].

### 2.3. In Vitro Reconstitution of proMMP-9∙CSPG Complexes Using Full-Length and Deletion Variants of proMMP-9

Previously, we showed that proMMP-9 formed SDS-stable and SDS-soluble complexes with purified SG and versican [39]. Because the vast majority of the secreted proteoglycan in the purified CSPG fraction was SG, we could assume that the *in vitro* reconstituted proMMP-9·CSPG complexes contained SG. Therefore, most of the *in vitro* reconstitution studies described below were with the purified CSPG, which we hereafter refer to as CSPG/SG. Some reconstitution experiments were performed with both CSPG/SG and SG purified from THP-1 cells, and as expected, the obtained results were identical. When we refer to the complexes obtained in the present work, they are called proMMP-9·CSPG/SG.

**Figure 3:**
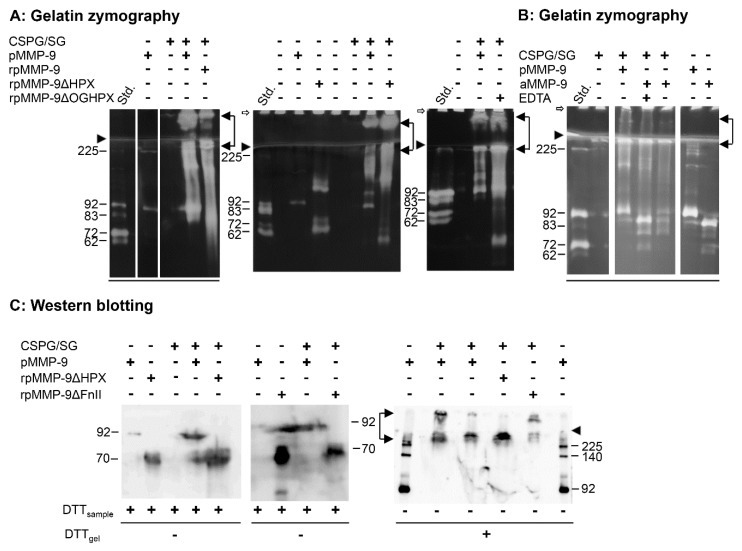
*In vitro* reconstitution of complexes between proMMP-9 and proMMP-9 deletion variants with CSPG/SG. Gelatin zymography (**A**,**B**) and Western blots (**C**) of proMMP-9 from THP-1 cells (pMMP-9), recombinant proMMP-9 (rpMMP-9), HPX-deleted proMMP-9 (rpMMP-9ΔHPX), FnII-deleted proMMP-9 (rpMMP-9ΔFnII), and trypsin-activated proMMP-9 from THP-1 cells (aMMP-9) and their complexes with CSPG/SG. Presence (+) and absence (−) of respective enzyme variants, CSPG/SG and EDTA (10 mM). The *in vitro* reconstitutions were performed as described in the Materials and Methods. The arrowheads indicate the border between the stacking and separating gel. Double arrows indicate the region of the SDS-stable complexes, one at the top of the separating gel and the other in the stacking gel. The other bands seen in the mixtures of CSPG/SG and the proMMP-9 variants are the SDS-soluble complexes. Open arrows indicate the bottom of the application wells, and the standard (Std.) is a mixture of MMP-9 (homodimer 225 kDa; pro 92 kDa; active 83 kDa) from THP-1 cells and MMP-2 (pro 72 kDa; active 62 kDa) from skin fibroblasts (**A**,**B**). In the Western blots (**C**), samples were run under either non-reducing conditions (DTT_sample_, −) or reducing conditions (DTT_sample_, +). Under reducing conditions, the samples were incubated with 0.1 M DTT prior to electrophoresis. When samples were run under non-reducing conditions, in order to detect the bound MMP-9 to CSPG/SG, prior to blotting, the gel was incubated for 1 h in 0.1 M DTT (DTT_gel_, +), in contrast to samples run under reducing conditions, as the gel was not incubated with DTT (DTT_gel_, −). The biotinylated protein ladder was used as the M_r_ marker in (**C**); lanes not shown. In (**A**,**B**), the same gel cut and pasted for clarity is shown by a line under the gels. Note that samples with CSPG/SG only do not contain any or only small amounts proMMP-9, and hence, no or eventually weak bands are seen.

Previous studies suggested that both the prodomain, the FnII module, and the HPX domain of proMMP-9 were involved in the formation of the proMMP-9∙CSPG complexes [37,38,39]. In the present study, purified CSPG/SG and SG from unstimulated THP-1 cells were used. Purified CSPG from unstimulated THP-1 cells (monocytes) contained no (or only a limited amount of) proMMP-9·CSPG, in contrast to CSPG isolated from PMA stimulated THP-1 cells (macrophages) [35]. Complexes formed by *in vitro* reconstitution with different MMP-9 variants along with CSPG/SG or SG were detected by gelatin zymography and Western blotting (Figure 3). The full-length variants of proMMP-9 (pMMP-9 and rpMMP-9) formed SDS-stable and SDS-soluble complexes with CSPG/SG (Figure 3A). To test if the prodomain was necessary for the complex formation between proMMP-9 and the CSPG/SG core protein, proMMP-9 purified from THP-1 cells was activated by trypsin. This generated an 83 kDa form of MMP-9 (Figure 3B) with ^88^F as the N-terminal amino acid [58], which we previously showed to be an active protease that degraded the fluorescence-quenched substrate McaPLGLDpaAR-NH_2_ [48]. As an active form of MMP-9 may cleave the CSPG/SG core protein, the *in vitro* reconstitution of the complex was performed in the presence and absence of the metalloproteinase inhibitor EDTA. As shown in Figure 3B, both SDS-stable and SDS-soluble complexes were formed, indicating that the presence of the prodomain was not necessary for complex formation. However, it appeared that the active form of MMP-9 could cleave the CSPG/SG core protein as less amount of complex was formed in the absence than in the presence of EDTA (Figure 3B).

Gelatin zymography showed that the HPX and OGHPX deletion variants of proMMP-9 (rpMMP-9ΔHPX and rpMMP-9ΔOGHPX) also formed SDS-stable and SDS-soluble complexes with CSPG/SG (Figure 3A). No such complexes were detected with the FnII deletion variants (rpMMP-9ΔFn, rpMMP-9ΔFnHPX, and rpMMP-9ΔFnOGHPX), using gelatin zymography as the detection method. This was the case both by using partly purified (S-200 and HS) and crude media containing rpMMP-9ΔFnII. Previously, it was shown that the specific activity against gelatin of active MMP-9 lacking the FnII module was only approximately 20% of the full-length variant [59]. This was similar to the active MMP-2ΔFnII, which showed activity against gelatin, which was approximately 10% of that of the active full-length variant of MMP-2 [60]. Therefore, Western blotting and an antibody detecting proMMP-9 were used to detect formed proMMP-9·CSPG/SG complexes (Figure 3C). *In vitro* reconstituted samples treated with DTT prior to electrophoresis showed that proMMP-9·CSPG/SG complexes were formed with most proMMP-9 variants, including rpMMP-9ΔFnII. However, no complexes were detected with the two deletion variants lacking both the FnII module and the HPX domain, i.e., rpMMP-9ΔFnHPX and rpMMP-9ΔFnOGHPX. Mixing and incubating the crude media containing these two deletion variants with purified full-length proMMP-9 and the CSPG/SG proteoglycan had no effect on the formation of the complex between the full-length proMMP-9 and CSPG/SG; neither did this crude media affect the binding of a preformed proMMP-9∙CSPG/SG complex to the Q-Sepharose column used to isolate the complex. This showed that the lack of complex formation between these two deletion variants of proMMP-9 and the CSPG/SG proteoglycan was neither due to impurities in the crude enzyme media that prevented the complex formation, nor the binding of the complex to the Q-Sepharose column, but due to the lack of both the HPX domain and the FnII module. In order to determine to which extent the formed proMMP-9·CSPG/SG complexes were of the SDS-stable or the SDS-soluble type, unreduced samples were applied to the SDS-PAGE gel. After electrophoresis and prior to blotting, the gel was incubated in 0.1 M DTT. The reason was that no CSPG or proMMP-9 bound to CSPG was transferred to the polyvinyl difluoride membrane from gels not treated with DTT prior to blotting [36]. In the presence of DTT, proMMP-9 was released from the CSPG. On the blot, MMP-9 was detected at a position corresponding to that seen in gelatin zymography [36,39]. Figure 3C shows that all samples containing either the HPX domain or the FnII module formed SDS-stable complexes. One complex was located in the stacking gel, while the other complex just entered the separating gel similar to that seen in gelatin zymography gels. Notably, by Western blotting, we never detected any SDS-soluble complexes, i.e., bands around 92 kDa for pMMP-9 and bands around 70 kDa for the truncated variants (rpMMP-9ΔHPX and rpMMP-9ΔFnII). This was in agreement with previous results of the isolated complex from THP-1 macrophages [36] and *in vitro* reconstituted complexes [39] and suggested a much larger formation of SDS-stable complexes than of SDS-soluble complexes. Furthermore, the intensities of SDS-stable and SDS-soluble complexes seen in gelatin zymography and the lack of detection of SDS-soluble complex in Western blots suggested that the auto-activation induced by the removal of SDS from the zymography gels was much less effective when proMMP-9 was bound to the CSPG/SG core protein. Another possibility was that the activity against gelatin was much lower for the activated MMP-9 bound to CSPG/SG than for the free MMP-9. If that was the case, two possibilities may explain a lower activity: 1. The activated MMP-9 bound to the CSPG/SG core protein had a lower specific activity against gelatin than the unbound active MMP-9. 2. The CSPG/SG core protein was an MMP-9 substrate, and hence, there would be a competition between the two substrates.

In conclusion, complexes between CSPG/SG and proMMP-9 were formed when proMMP-9 contained either both the HPX and FnII domains or only one of these domains. However, when both domains were absent, no complexes were formed. This indicated that the main interactions of the CSPG/SG core protein with proMMP-9 in these complexes were with the HPX domain and the FnII module, although independently of each other. Even though it appeared that the pro-, catalytic-, and OG domains were not involved in the complex formation between proMMP-9 and the CSPG/SG core protein, it could not be excluded that these domains may have an effect on the kinetics of the complex formation or even be involved in binding. In the latter case, the interaction was not strong enough to form the proMMP-9·CSPG/SG complex.

### 2.4. In Vitro Reconstitution of proMMP-9∙Serglycin Core Protein Complexes Lacking CS Chains

**Figure 4:**
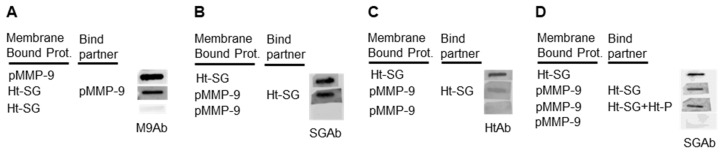
ProMMP-9∙Ht-SG complex formation. Fifty nanograms of pMMP-9 and 200 ng Ht-SG were added to a polyvinyl membrane using a slot-blot apparatus as described in the Methods. Briefly, the membranes were thereafter washed in TBST, incubated in blocking buffer containing milk powder (BB) for 1 h. Membranes with added pMMP-9 and Ht-SG were cut out, and membrane pieces containing bound pMMP-9 were incubated with either blocking buffer (BB), 5 ng/µL (0.29 μM) Ht-SG (in BB), or 0.29 μM Ht-SG containing 0.87 μM His-tagged peptide (Ht-P). Membrane pieces with bound Ht-SG were incubated with either BB or 50 ng/µL pMMP-9 (in BB) for 2.5 h at room temperature. After washing three times with TBST, the membrane pieces were incubated for 24 h at 4 °C with either antibodies against proMMP-9 (**A**), serglycin (**B**,**D**), or serglycin His-tag (Ht Ab) (**C**). The various membranes were thereafter washed with TBST, incubated with HRP-conjugated secondary antibody, washed with TBST, and subjected to antibody detection by the Luminol reagent and the Fujifilm LAS-4000 system. Each slot under A, B, C, and D, respectively, was from the same exposure time and exposed to the CCD camera at the same time.

To verify that pMMP-9 could bind to the core protein of SG, binding studies were performed using pMMP-9 purified from THP-1 cells and a commercial recombinant human His-tagged SG (Ht-SG) produced in Escherichia coli. Ht-SG contained a 25 amino acid long N-terminal His-tag (MGSSHHHHHHSSGLVPRGSHMGSHM) instead of the signal peptide domain (predomain) and lacked GAG chains. In these binding studies, either the pMMP-9 or Ht-SG was bound to a polyvinyl difluoride membrane using a slot blot apparatus. The different membrane slots were cut out and blocked with milk powder as for Western blotting. Thereafter, relevant membrane slots were incubated with either pMMP-9 or Ht-SG. The slots were then washed and incubated with antibodies against either pMMP-9, SG, or the His-tag as described in the Materials and Methods. As shown in Figure 4A, the MMP-9 antibody (M9Ab) detected pMMP-9 and pMMP-9 bound to the Ht-SG, but not Ht-SG. Similarly, the antibodies against SG (SGAb) and the His-tag antibody (HtAb) detected Ht-SG and Ht-SG bound to pMMP-9, but not pMMP-9 (Figure 4B,C). Experiments were performed to determine whether the binding of MMP-9 was to the SG core protein or to the N-terminal His-tag peptide. Membranes with bound pMMP-9 were incubated with either Ht-SG or a mixture of Ht-SG and a His-tag peptide (Ht-P), which was identical to the 25 N-terminal amino acids in the Ht-SG. The SG antibodies (SGAb) were used to detect binding of Ht-SG to proMMP-9. The Ht-P did not inhibit the binding of Ht-SG to pMMP-9 (Figure 4D). This showed that pMMP-9 bound to the core protein of SG, and not to the His-tag peptide part of Ht-SG.

In conclusion, these experiments showed that proMMP-9 and the SG core protein could bind and form a complex. However, it was not shown if the formed complex was of the SDS-stable or -soluble type. They also showed that the MMP-9 antibody did not detect the SG core protein, and the antibodies against SG did not detect proMMP-9.

### 2.5. Peptide Arrays

To gain insight into which parts in the SG core protein, the MMP-9HPX domain and MMP-9FnII module, were involved in the formation of the proMMP-9∙SG complex, peptide arrays of these three proteins were synthesized. The first set consisted of 20-mer peptides bound on a cellulose membrane, where each peptide was obtained by a peptide walk along the entire protein sequences with two amino acid intervals. A second set of mutated peptide arrays based on the results from the first set was produced as described in the Materials and Methods. Control experiments against both sets of peptide arrays were conducted both with primary and secondary antibodies to the binding protein (proMMP-9 and SG) to rule out binding caused by the antibodies. Therefore, the data presented were only for those arrays with no interference from unspecific binding of the antibodies. Unspecific binding of antibodies prohibited the determination of interactions between the peptide and the anticipated binding partners. Ht-SG was used for the binding studies of SG to the peptide arrays of the MMP-9HPX domain and the MMP-9FnII module, while only the three proMMP-9s, pMMP-9, rpMMP-9, and rpMMP-9ΔHPX purified to homogeneity, were used for the binding studies of proMMP-9 to the peptide arrays of SG.

#### 2.5.1. Binding of proMMP-9 to Serglycin Peptide Arrays

**Figure 5:**
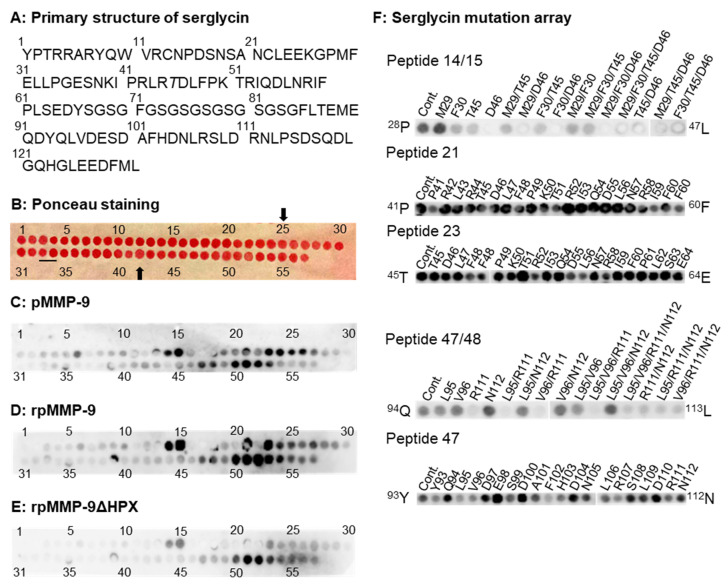
Binding of proMMP-9 variants to SG peptide arrays. (**A**) Primary structure of SG. (**B**) Ponceau staining of the 20-mer peptides bound on a cellulose membrane, where each peptide was obtained from a peptide walk along the entire SG sequence with an interval of two amino acids. For simplicity, every fifth peptide in the array is numbered, and the arrows show the starting and ending of peptides containing the GAG binding amino acids Ser-Gly (amino acids 67–84) in the center of the core protein; the two peptides that contain the entire 8 Ser-Gly repeats are underlined. (**C**) Binding of proMMP-9 purified from THP-1 cells (pMMP-9) to peptides in the SG core protein. (**D**) Binding of full-length recombinant proMMP-9 (rpMMP-9) to peptides in the serglycin core protein. (**E**) Binding of recombinant HPX truncated proMMP-9 (rpMMP-9ΔHPX) to peptides in the SG core protein. (**F**) Binding of pMMP-9 to a SG peptide arrays containing mutated amino acids based on the interpretation of the binding to peptides observed in Figure 5C,D. Two different arrays were performed where either (1) a combination of the predicted four possible amino acids from the binding pattern obtained in Figure 5C,D were mutated or (2) where every amino acid in the peptide was mutated as described in the Materials and Methods. Shown above each array are: (1) the number of the peptide in Figure 5C,D that was used for the mutation screen and (2) the name and number of the parent amino acid mutated. Shown also is the position of the N- and C-terminal amino acid of the 20-mer peptides. The un-mutated control peptide is labelled with Cont.

The human SG core protein consists of 131 amino acids [21]. The mid-section contains 8 Ser-Gly repeats (amino acids 67–84) to which GAG chains are attached (Figure 5A), giving a peptide array of 57 spots (Figure 5B). Control experiments with primary and secondary antibodies revealed that they did not bind to any of the peptides in the array. However, in one of the mutated peptide arrays, the primary antibody against MMP-9 did bind to peptide 52 and several of the mutated variants of the peptide (Figure S2). As seen in Figure 5, all proMMP-9 variants bound amino acid sequences located both N-terminal and C-terminal of the GAG-attachment sites.

The two full-length forms of proMMP-9 (pMMP-9 and rpMMP-9) showed an almost identical binding pattern for the SG peptide array (Figure 5C,D). As a control, pMMP-9 was incubated with another identical array (Figure S3) resulting in small differences in the intensity of the signals. This could be due to small differences in the produced batches of the peptide arrays. Binding of proMMP-9 to the region N-terminal to the SG GAG-attachment sites (peptide 6) indicated that the entire sequence from ^11^V to ^30^F was required for binding, as the mutation array where the ^11^VR and ^29^MF amino acids were replaced did not prevent binding. ProMMP-9 also bound to peptides 14 and 15 (Figure 5C,D, Figure S3). A mutation array where the putative interacting amino acids ^29^MF and ^45^TD were replaced (Figure 5F) revealed that changing ^46^D to A prevented the binding of proMMP-9. However, changing ^29^M to G did not affect binding. Replacing ^30^F and ^45^T by A and V, respectively, resulted in reduced binding of proMMP-9 to the peptide. The interactions of proMMP-9 with these two peptides, but not peptides 13 and 16, suggested a binding to the side chain of ^46^D and likely to the side chains of ^30^F and ^45^T, as well as the main chain amide of the latter two amino acids.

ProMMP-9 also seemed to bind peptides 21–27. Mutated peptide arrays of peptides 21 and 23 revealed that no single mutation prevented binding (Figure 5F), suggesting that several side chains from ^41^P to ^64^E were involved in binding. In these peptides, mutation of ^46^D to A did not affect binding.

ProMMP-9 also bound to peptides 47 and 48 located at C-terminal to the GAG attachment sites (Figure 5C,D, Figure S3). Mutation arrays of these two peptides suggested that several of the side chains from ^93^Y to ^114^P must be involved in the binding as none of the single amino acid mutations prevented binding, but only appeared to reduce binding (Figure 5F).

ProMMP-9ΔHPX had similar binding patterns as the two full-length enzymes, but did not bind peptide 21 (Figure 5E).

#### 2.5.2. Binding of Serglycin to MMP-9 FnII Peptide Arrays

**Figure 6:**
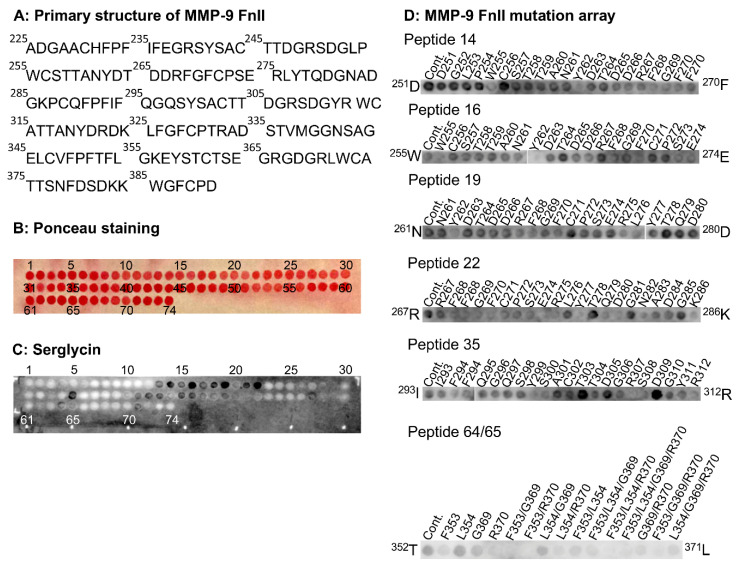
Binding of SG to MMP-9 FnII module peptide arrays. (**A**) Primary structure of the FnII module. (**B**) Ponceau staining of the 20-mer peptides bound on a cellulose membrane, where each peptide was obtained from a peptide walk along the entire MMP-9 FnII sequence with an interval of two amino acids. For simplicity, every fifth peptide in the array is numbered. (**C**) Binding of recombinant Ht-SG to peptides in the FnII module. (**D**) Binding of recombinant Ht-SG to FnII peptide arrays containing mutated amino acids based on the binding obtained in Figure 6C. Two different arrays were performed where either (1) a combination of the predicted four possible amino acids from the binding pattern obtained in Figure 6C were mutated or (2) where every amino acid in the peptide was mutated as described in the Materials and Methods. Shown above each array is: (1) the number of the peptide in Figure 6C that is used for the mutation screen and (2) the name and number of the parent amino acid mutated. Shown also is the position of N- and the C-terminal amino acid of the 20-mer peptides. The un-mutated control peptide is labelled with Cont.

The FnII module in proMMP-9 contained 166 amino acids (^225^A-^390^D; Figure 6A), giving a peptide array of 74 spots (Figure 6B). Control experiments with primary and secondary antibodies revealed that they did not bind to any of the peptides in the array. Figure 6C shows that SG bound to several peptides; however, mutated peptide arrays revealed that the binding to peptides 41–43, 45, 46, 49, 57, and 71 appeared to be artefacts (Figure S4). Therefore, we concluded that SG bound to peptides 13–22 (^249^R-^286^K), 35 (^293^I-^312^R), 64 (^351^F-^370^R), and 65 (^353^F-^372^W). Peptides 13–22 comprised the FnII repeat 1, peptide 35 the first part of repeat 2, and peptides 64/65 the first part of repeat 3.

To test which amino acids in the FnII module were involved in the binding of SG to peptides 13 to 22, full mutation arrays of peptides 14, 16, 19, and 22 (Figure 6D) were performed. Mutation of amino acids ^255^W and ^262^Y to alanine seemed to prevent or weaken the binding of SG to peptides 14 and 16. In peptide 19, it appeared that only mutation of amino acid ^262^Y prevented or weakened the binding. In peptide 22, mutations of several amino acids appeared to weaken binding, while mutation of amino acids ^274^E and ^277^Y seemed to prevent binding. In peptide 35, mutations of several amino acids seemed to weaken binding. Only the mutation of amino acid ^307^R appeared to prevent binding. A selected mutation peptide array of peptide 64/65 suggested that amino acids ^353^F and ^370^R were involved in binding of SG.

#### 2.5.3. Binding of Serglycin to MMP-9 HPX Peptide Arrays

**Figure 7:**
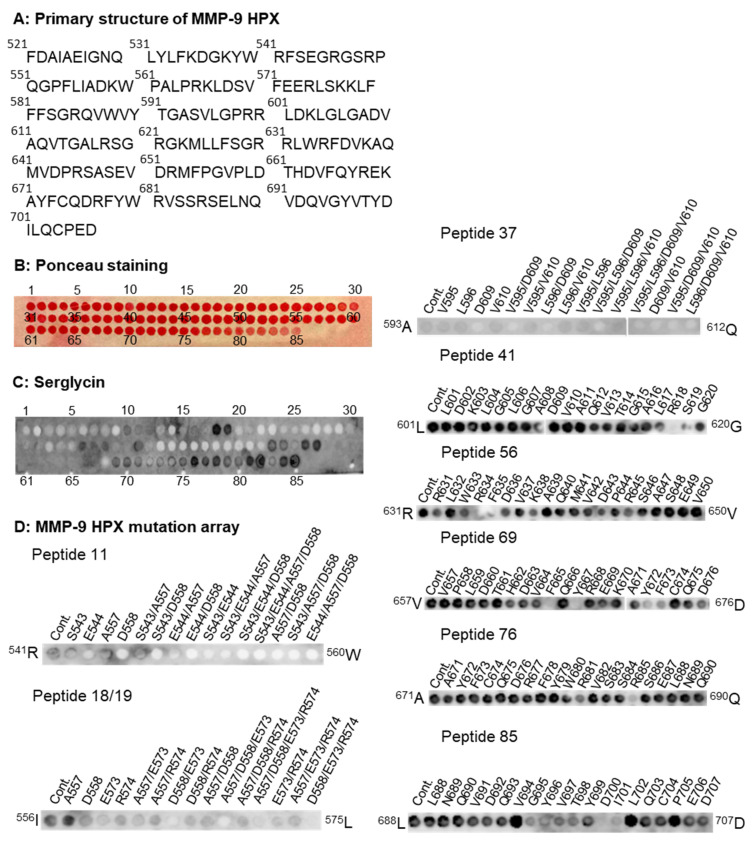
Binding of SG to MMP-9 HPX peptide arrays. (**A**) Primary structure of the HPX domain. (**B**) Ponceau staining of the 20-mer peptides bound on a cellulose membrane, where each peptide was obtained from a peptide walk along the entire MMP-9 HPX sequence with an interval of two amino acids. For simplicity, every fifth peptide in the array is numbered. (**C**) Binding of recombinant Ht-SG to peptides in the HPX domain. (**D**) Binding of recombinant Ht-SG to HPX peptide arrays containing mutated amino acids based on the binding obtained in Figure 7C. Two different arrays were performed where either (1) a combination of the predicted four possible amino acids from the binding pattern obtained in Figure 7C were mutated or (2) where every amino acid in the peptide was mutated as described in the Materials and Methods. Shown above each array is: (1) the number of the peptide in Figure 7C that is used for the mutation screen and (2) the name and number of the parent amino acid mutated. Shown also is the position of N- and the C-terminal amino acid of the 20-mer peptides. The un-mutated control peptide is labelled with Cont.

The MMP-9 HPX domain contains 187 amino acids (^521^F-^707^D; Figure 7A), giving a peptide array of 85 spots (Figure 7B). Control experiments with primary and secondary antibodies revealed that they did not bind to any of the peptides in the array. Figure 7C shows that SG bound to several peptides. Control experiments with mutated peptide arrays (Figure S5) revealed that the binding to peptides 36, 53, 57, 78, and 82 appeared to be artefacts, as the primary SG antibodies bound to these peptides. The binding to peptides 15–17 also appeared as artefacts, since the SG did not bind to the mutation array of peptide 16. Therefore, we concluded that SG bound to the following peptides; 10–12, 18, 19, 37–38, 41, 56, 69–71, 76, 84, and 85. This suggested that the binding of SG involved the end of blade 1 (peptides 10–12 and 18/19), the end of blade 2 (peptides 37, 41), β-strands 2–4 blade 3 (peptide 56), the end of blade 3, the beginning of blade 4 (peptides 69–71), and most of the end of blade 4 (peptides 76, 84, 85).

To determine which amino acids were involved in binding of SG to peptides 10–12 of the HPX domain (Figure 7C), a selected mutation array of peptide 11 was performed (Figure 7D). This suggested that ^544^E and ^558^D were involved in the binding of the SG core protein. A selected mutation array of peptide 18/19 (Figure 7D) suggested that the mutation of ^573^E weakened the binding, while the mutation of amino acid ^609^D seemed to prevent the binding of SG to peptide 37 (Figure 7D). Peptide 41 was at the end of blade 2 and the beginning of blade 3, and the mutation array of this peptide suggested that the mutation of ^618^R to alanine prevented binding and, so, maybe also the mutation of ^619^S (Figure 7D). However, the mutation of ^609^D did not affect the binding of the SG core protein to peptide 41. None of the mutations of peptide 56 appeared to prevent binding (Figure 7D), suggesting that most of the amino acids in this peptide were involved in the interaction. The peptide 69 mutation array revealed that the mutation of ^665^F and ^667^Y seemed to prevent binding, while the mutation of ^672^Y and ^673^F seemed to weaken binding (Figure 7D). SG binding to peptide 76 seemed to be prevented or weakened by a mutation of amino acid ^685^R to alanine (Figure 7D). A mutation array of peptide 85 indicated that the mutations of amino acids ^700^D and ^701^I prevented binding (Figure 7D). Due to the binding of the primary antibody to the peptides with the following mutated amino acids (^694^V, ^699^Y, ^702^L, and ^705^P) in peptide 85 (Figure S5), it was not possible to conclude whether these mutations affected binding or not.

The peptide arrays suggested that proMMP-9 bound to residues in SG located both at the N- and C-terminal to the CS attachment sites. The arrays also suggested that SG bound to several parts of both the FnII module and the HPX domain. The *in vitro* reconstitution experiments revealed that either the FnII module or the HPX domain must be present in order to form an SDS-stable proMMP-9∙CSPG/SG complex. Several questions arose: (1) Can the FnII module or the HPX domain interact with both the N- and C-terminal part of SG at the same time? (2) Can one SG molecule at the same time bind to all sites suggested by the peptide arrays in either the FnII module or the HPX domain, or are several complexes possible with slightly different orientations of the SG? (3) Can one SG molecule bind to the FnII module and the HPX region at the same time? We used protein modelling, docking, and molecular dynamics (MD) simulations in an attempt to answer these questions.

### 2.6. Docking and Molecular Dynamic Studies of the Interaction of Serglycin with the MMP-9 FnII Module and HPX Domain

Docking and MD simulations of the SG core protein with the proMMP-9 HPX domain and the proMMP-9 FnII module were performed in order to predict the interaction modes of SG with the HPX domain and the FnII module.

#### 2.6.1. Protein Modelling and Protein-Protein Docking

X-ray crystallography of the cloned MMP-9 HPX domain showed a dimeric structure [30]. To test the performance of the BioLuminate program, the HPX_B_ subunit of the dimeric structure was docked to the HPX_A_ subunit. The docking gave 67 different poses, and the best pose was selected based on its structural similarity with the dimeric X-ray structure. Structural superimposing with the X-ray structure showed that the docked complex had an almost complete overlap with the X-ray structure dimer (Figure S6.1 in S6). We compared the position of the amino acids causing strong interactions between the two subunits in the X-ray structure dimer [30] with the corresponding amino acids in the docked complex. Cha et al. [30] reported that ^707^D in HPX_A_ formed an ionic interaction with ^677^R in HPX_B_, while the hydrophobic cluster ^699^Y, ^696^Y, and ^694^V in HPX_A_ interacted with the hydrophobic cluster ^696^Y, ^678^F, and ^694^V in HPX_B_. An interaction between the side chains of ^651^D in HPX_A_ with ^685^R in HPX_B_ was also reported. Figure S6 shows that these residues in the docked complex were overlapping with the X-ray structure dimer. These docking results indicated that protein-protein docking also could be useful for obtaining further information about the interactions between the SG core protein and proMMP-9.

**Figure 8:**
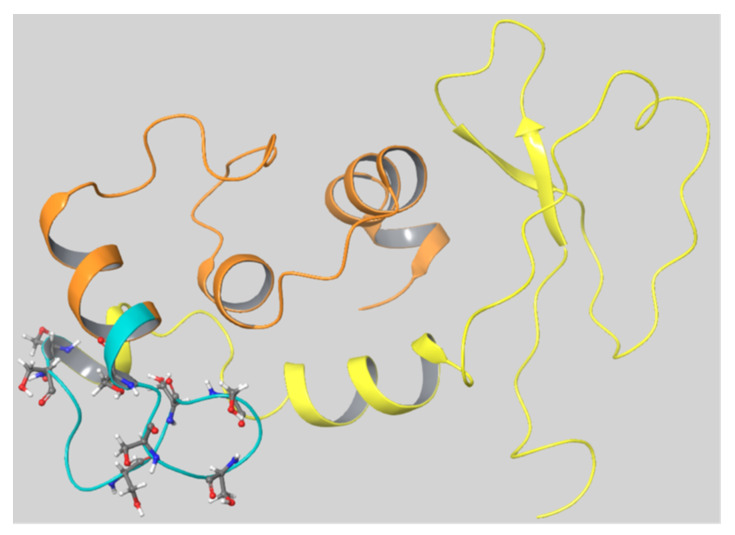
Predicted structure of the SG core protein. The prediction was based on the primary structure of the SG core protein using the Phyre^2^ program as described in the Materials and Methods. The N-terminal amino acids from 1–66 are labeled yellow; the amino acid stretch from 67–83, which contains the Ser-Gly repeats that bind the CS chains, are labeled in light blue; and the C-terminal stretch from amino acids 84 to 131 are labeled in orange. Shown are the eight serine residues that are the putative CS chain attachment sites with nitrogen in blue, carbon in grey, oxygen in red, and hydrogen in white.

As there was no known 3D structure of the SG core protein, a homology model of the protein was constructed using the Phyre^2^ program as described in the Materials and Methods. The program predicted models of SG based on homology with several known protein structures and the model based on template 6ewvA (PDB id: 6ewv) had the highest confidence (59%). The obtained model showed a hairpin-like structure where the Ser-Gly repeats that bound the CS chains formed a loop and the regions C- and N-terminal to the Ser-Gly repeats appeared to join (Figure 8).

**Scheme 1:**
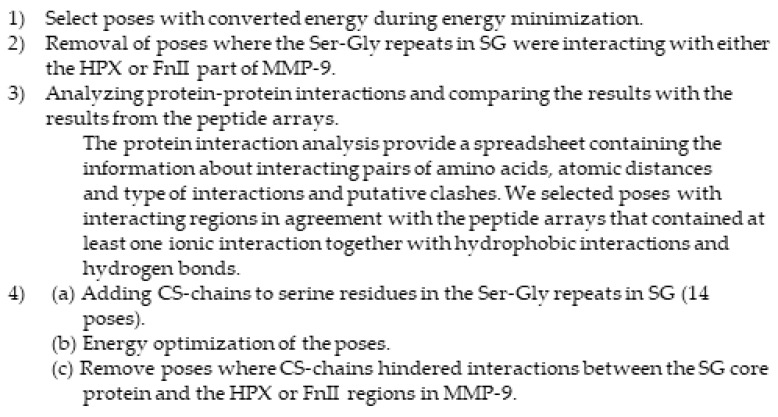
Steps for selecting the best docking poses for MD.

The SG docking with FnII resulted in 130 poses, while the docking with HPX gave 77 poses. The best poses were selected using four steps (Scheme 1).

Step (1) gave 30 poses from the docking of SG and FnII and 19 poses from the docking of SG and HPX. Step (2) reduced the number of poses with FnII to 7 and with HPX to 12. Step (3) resulted in three poses from the docking of SG with FnII and 11 poses from the docking of SG with HPX. In Step (4a), CS chains were added to five of the serine residues in the Ser-Gly repeats of SG as described in the Materials and Methods. To which of these eight serine residues they should be added and the exact number of CS chains are, to our knowledge, unknown. It has been suggested that four, but not more than six CS chains are attached (Prof. S.O. Kolset and Prof. K. Prydz, University of Oslo, Norway, personal communication, and [43]). N-terminal sequencing of SG from U-937 cells gave the following sequence, SEDYXXXGFG [61]. This suggested that CS chains were attached to the two first serine residues in the Ser-Gly repeat, i.e., ^67^S and ^69^S (see Figure 5 for the sequence), which was the reason why we added CS chains to these two residues. In addition, we added CS chains to ^73^S, ^77^S, and ^83^S. Addition of CS chains to the other serines of the Ser-Gly repeat gave steric clashes, and CS chains were not attached to these serine residues in further studies. Step 4b and c gave two poses from the FnII docking and four from the HPX docking that were used for MD simulations.

#### 2.6.2. Molecular Dynamics Simulation

MD simulations were performed for the two docking complexes of SG with FnII, the four complexes of SG with HPX. Each complex was simulated twice. In addition, two simulations were performed with the docked HPX dimer and one with the X-ray dimer. The motivation for the MD simulations was to check the stability of the interactions in the docked complexes and if the simulations could induce additional protein-protein interactions in the complexes. First, we performed MD simulations of the HPX X-ray dimer and the best pose from the docking of HPX_B_ to the HPX_A_ subunit and compared the results. These MD simulations confirmed a large similarity between the HPX dimer X-ray structure and the docked complex (Supplement 1, Figure S6). These results suggested that docking and MD simulations were valuable approaches for studying interactions of the SG core protein with the FnII module and the HPX domain of MMP-9.

Based on analysis of distance plots generated for interacting amino acids during MD simulations of SG with FnII and HPX, the most stable poses were selected as the most probable structure of the complexes. This resulted in one putative model for the interaction of SG with FnII and two putative models for the interaction of SG with HPX. These models are described in the following. The selected amino acid pairs of interactions for all three complexes are summarized in Table 1, Table 2 and Table 3 along with the information from their respective peptide arrays. Results from MD simulations are also shown in Figures S7–S9.

#### 2.6.3. Binding of Serglycin with FnII

**Figure 9:**
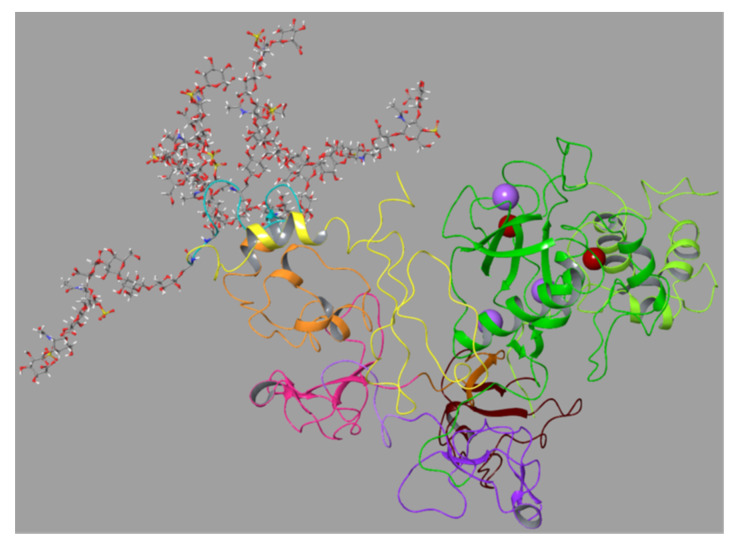
SG docked to the FnII module in the catalytic domain of proMMP-9. The SG core protein was docked into the FnII module of proMMP-9 using the BioLuminate program as described in the Materials and Methods. The pro- and catalytic-domain in MMP-9 are labelled in light and dark green, respectively. The FnII repeat 1 is purple; repeat 2 is pink; and repeat 3 is brown. The two zinc atoms are labelled dark red, and the three calcium atoms are labelled purple. The SG core protein is labelled as in Figure 8. The carbon atoms in the CS chains are grey, and the sulfur atoms are yellow.

The two MD simulations of each complex resulted in almost identical dynamic movements of the interacting amino acids indicating the reproducibility of the simulations. For the model considered as the most probable, most of the amino acids in the FnII module that interacted with SG were found in the second repeat, while two out of fourteen total amino acids were located between the first and second repeat (Figure 9). A detailed description of the interactions between the SG core protein and the MMP-9 FnII module is found in Table 1, Supplement 2, and Figure S7. In SG, almost all the amino acids that interacted with FnII were in flexible loops with the majority in the core protein located at the C-terminal of the Ser-Gly repeat. The MD simulations indicated that the complex was held together by a combination of ionic and hydrophobic interactions, as well as hydrogen bonds (Table 1 and Figure S7). This model showed one ionic bond between ^280^D in FnII and ^42^R in SG that was initially stable, but after 50 ns, the distance started to increase with time. One stable ionic interaction was observed, and this was between ^162^R in the catalytic domain of MMP-9 with ^46^D in SG.

Binding of rpMMP-9ΔHPX to the SG peptide array indicated that the FnII module of proMMP-9 could bind amino acids located at both N- and C-terminals for the Ser-Gly repeats (Figure 5). Protein modelling of SG, docking, and MD simulations of the formed complex between SG and the FnII module in proMMP-9 suggested that the FnII module could simultaneously bind amino acids located both at the N- and C-terminal to the Ser-Gly repeats in SG. This also suggested that it was not possible for one SG molecule to bind two proMMP-9 molecules at the same time, i.e., one at both sides of the Ser-Gly repeats. SG also interacted with amino acids in the first and second repeat of the FnII module. Docking and MD also indicated that amino acids located in peptides 41–43 in the peptide array were involved in binding, although the peptide array studies indicated that this binding might be an artefact. The peptide array suggested that peptides 64 and 65 could bind the SG core protein, but these peptides were not predicted by docking and MD simulations. The docking and MD simulation studies indicated that the SG core protein covered a large part of the FnII surface and that the proMMP-9∙SG complex formed by the FnII module was a result of only one complex and hence only one interacting region.

In proMMP-9·CSPG isolated from PMA-treated THP-1 cells, the CSPG core protein bound and hid epitopes in the FnII module of proMMP-9 that were involved in the binding of gelatin [38]. Only a small fraction of the proMMP-9·CSPG complex (15–35%) bound gelatin, while the majority of the proMMP-9·CSPG complex did not. This binding was much weaker than the binding of gelatin to proMMP-9. These results indicated that the interaction between proMMP-9 and the core protein(s) in the CSPG induced a new binding site for gelatin in some of the formed complexes. When gelatin bound to proMMP-9·CSPG complexes, proMMP-9 and proMMP-9·CSPG appeared to bind different epitopes in gelatin. This was also the case for the *in vitro* reconstituted proMMP-9·CSPG/SG SDS-stable and SDS-soluble complexes [39]. Furthermore, increasing concentrations of gelatin along with CSPG and proMMP-9 in the *in vitro* reconstitution assay resulted in decreasing formation of both the SDS-stable and SDS-soluble complexes [39]. Collier et al. [50] performed alanine scanning mutagenesis and functional analysis of gelatin binding to the FnII module in MMP-9. These studies indicated that gelatin mainly bound to the second FnII repeat and that the amino acids ^307^R, ^309^D, ^319^N, ^320^Y, and ^323^D were critical for binding. In the peptide array (Figure 6), these amino acids were located in the peptide spots 35 and 41–46, which all bound SG. In the docking and MD simulation studies, amino acids between ^306^G to ^328^F in the FnII module were involved in binding of the SG core protein. This suggested that there were overlapping epitopes in the FnII module to which both SG and gelatin bound. Therefore, the results of the peptide arrays and the MD simulation studies were in agreement with the *in vitro* reconstitution experiments showing that gelatin inhibited the formation of the proMMP-9∙CSPG/SG complex [39]. Furthermore, the results were also in agreement with the alanine scanning study of Collier et al. [50], who determined where gelatin bound in the FnII module.

#### 2.6.4. Binding of Serglycin with HPX

Based on interaction patterns, we selected two models as possible for the HPX-SG interactions. In Model 1, HPX was involved entirely with the region of SG located at the N-terminal to the Ser-Gly repeats, while in Model 2, HPX mainly interacted with the region at the C-terminal to the Ser-Gly repeats.

**Figure 10:**
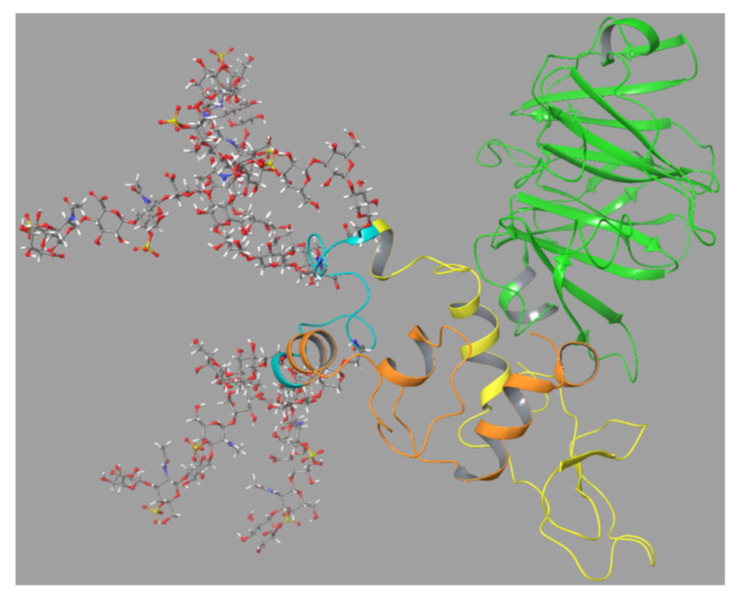
SG docked to the MMP-9-HPX domain, Model 1: The SG core protein was docked into the MMP-9-HPX domain using the BioLuminate program as described in the Materials and Methods. The HPX domain is labelled in light green, and the SG core protein with attached CS chains is labelled as in Figure 8 and Figure 9.

Model 1: The majority of the amino acids in the HPX domain that interacted with SG were from the fourth blade, while the remaining were from the third blade (Figure 10). A detailed description of this model is found in Table 2, Supplement 3, and Figure S8. The MD simulations indicated that a combination of ionic and hydrophobic interactions, as well as hydrogen bonds was responsible for the interaction between SG and HPX.

**Figure 11:**
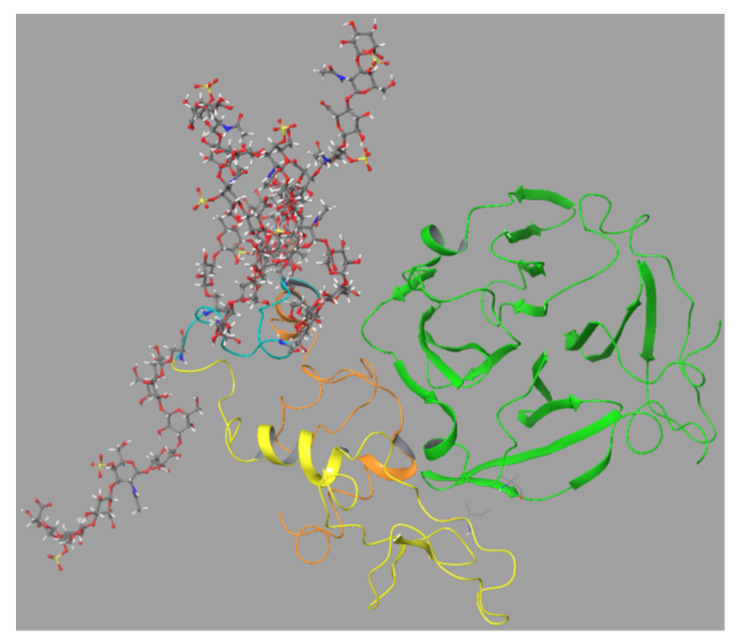
SG docked to the MMP-9-HPX domain, Model 2: The SG core protein was docked into the MMP-9-HPX domain using the BioLuminate program as described in the Materials and Methods. The HPX domain is labelled in light green, and the SG core protein with attached CS chains is labelled as in Figure 8 and Figure 9.

Model 2: Most amino acids of HPX that interacted with SG were from the third blade, together with some from the fourth blade (Figure 11). A detailed description of the model is given in Table 3, Supplement 4 and Figure S9.

Three positively charged HPX amino acids and two negatively charged SG amino acids formed a cluster of ionic interactions (^623^K, ^634^R, and ^645^R in HPX, ^97^D and ^98^E in SG). These amino acids were very dynamic where at least one of the negatively charged groups was always close (<2 Å) to one of the positively charged groups during MD (Figure S9.1 in S9). The other interactions were a combination of hydrogen bonds and hydrophobic interactions.

Both models seemed to contain all necessary interactions for an SDS-stable and hence a reduction-sensitive complex. Both models contained overlapping regions in the HPX domain that corresponded to the peptide spots 69–71 and 76–85 in the peptide array (Table 2 and Table 3, Figure 7), although there were different regions in the SG core protein that interacted with HPX in the two models. It is not possible to state that one of the models was more probable than the other model. In the *in vitro* reconstituted complex, rpMMP-9ΔFnII·CSPG/SG, the proteoglycan core protein bound only to the HPX domain in MMP-9. If both models corresponded to the in vitro formed complex, one must anticipate that there would be a competition between the two possible interaction modes of HPX and SG. This may have an effect on the amount of formed complex, depending on the kinetics and if there was a specific sequential binding of interacting regions. In the case of the complex formed with the full-length proMMP-9, two probable interaction regions in the HPX domain may also be of importance if the SG core protein can be sandwiched between the HPX domain and the FnII module due to the flexibility of the full-length proMMP-9 enzyme.

#### 2.6.5. Molecular Dynamics Simulations of Full-Length proMMP-9 with and without Bound Serglycin

One question we also wanted to address was if one SG molecule could simultaneously bind both the FnII module and the HPX domain in proMMP-9 and, if so, which parts of the two molecules were involved. The MMP-9 structure was very flexible due to the long hinge region, and the structure of the entire proMMP-9 was not known. Therefore, we tried to model the entire proMMP-9 molecule (^29^V-^707^D). The sequence used in these experiments was without glycosylation of the MMP-9 hinge region (OG domain). The human proMMP-9 sequence (^29^V-^707^D) was obtained from UniProt database, Identification Code P14780. The homology modelling option of the Schrödinger software was used to obtain a 3D model of the full-length proMMP-9. This resulted in a model with an N-terminal part that could be directly overlaid with the X-ray structure of mini-MMP-9, i.e., the prodomain and the catalytic domain with the FnII module (PDB ID:1l6j) and the C-terminal part with the X-ray structure of the HPX domain (PDB ID:1itv). Previously, it was shown by SAXS in combination with AFM that proMMP-9 was very flexible and that the two globular domains, i.e., the catalytic domain and the HPX domain, were either located close to each other or at a long distance from each other [29]. Therefore, we used the proMMP-9 model as the starting structure for a 500 ns MD simulation in order to determine whether the simulation reflected the observations from the combined SAXS and AFM study. The result was that the two globular parts moved to and from each other due to the very flexible hinge region (Movie 1). Figure S10 shows the first and last frame of the MD simulation. In the beginning of the MD, the two globular domains were at a close distance, and we designated this as the closed conformation (Figure S10). This suggested that it should be possible to test if SG could be stably sandwiched between the FnII module and the HPX domain and if SG could stably bind to either the FnII module or the HPX domain during the MD.

The SG (without CS chains) was linked to the FnII module as suggested by the best docking mode as described in Section 2.6.3, Figure 9. This was performed with the closed conformation of proMMP-9 as seen in Figure S10. This model of proMMP-9 with the bound SG core protein was then applied for a 500 ns MD simulation. This resulted in the SG sandwiched between the FnII module and the HPX domain after approximately 20–30 ns. This structure remained stable for the rest of the MD (Movie 2, Figure S11). Amino acids in SG corresponding to those in peptides 40–57 in the peptide array interacted with amino acids in MMP-9FnII corresponding to peptides 19–28 and 32–42 in the peptide array (Table 1). SG amino acids corresponding to peptides 7–18 in the peptide array interacted with amino acids in MMP-9HPX corresponding to peptides 59–68 and 78–85 in Table 2 and Table 3). Thus, the interactions seen between the full-length MMP-9 and SG in this MD simulation corresponded well with the results from the peptide arrays and dockings.

We also investigated if the SG core protein could interact with proMMP-9 by placing SG between the FnII module and the HPX domain when the two domains were at different distances from each other. MD simulations were performed for 100 ns. In two of the cases (Movies 3 and 4, Figures S12 and S13), the SG core protein was initially at an equal distance from the FnII module and the HPX domain. In the third case (Movie 5, Figure S14), the SG core protein was initially placed closer to the FnII module. In all cases, the Ser-Gly repeats in SG were pointing away from proMMP-9. When SG was positioned between the FnII module and the HPX domain in the MMP-9 structure obtained after 15 ns of the MD shown in Movie 1, the SG core protein was sandwiched between the FnII module and the HPX domain after 10 ns. The eight Ser-Gly repeats were pointing away from MMP-9. After that, the complex remained stable during the rest of the MD (Movie 3, Figure S12). Amino acids in SG corresponding to those in peptides 1–19 in the peptide array interacted with amino acids in MMP-9FnII corresponding to peptides 19–28 in the peptide array (Table 1). SG amino acids corresponding to peptides 20–29 and 40–52 in the peptide array interacted with amino acids in MMP-9HPX corresponding to peptides 4–13 and 74–85 (Table 2 and Table 3). Thus, the interactions seen between the full-length MMP-9 and SG in this MD simulation corresponded well with the results from the peptide arrays and docking studies.

In the conformation obtained after 32 ns of the MD shown in Movie 1, the SG was located between the FnII module and the HPX domain in the MMP-9 structure followed by a 100 ns MD (Movie 4, Figure S13). After 10 ns, the SG core protein started to move towards the HPX domain and thereafter remained stably bound during the rest of the MD. Between 10–50 ns, the amino acids in the HPX domain corresponding to peptides 10–20 and 28–38 of the peptide arrays interacted with the amino acids in SG corresponding to peptides 10–29 and 56–57 of the peptide arrays (Table 2 and Table 3). This fit partly with the peptide arrays (Figure 5 and Figure 7). From 50–100 ns, the amino acids in the MMP-9 HPX corresponding to peptides 5–14 in the peptide array interacted with the amino acids in SG corresponding to peptides 36–45 in the peptide array (Table 2 and Table 3). None if these interactions were seen in the peptide arrays. After 60 ns, the major part of N-terminal of SG also interacted with the OG domain in the MMP-9, which remained interacting throughout the MD as the other interactions.

In the third case, the SG core protein was placed close to the FnII module in the 32 ns frame from Movie 1. The SG was oriented in a position based on the SG from the best docked model with the FnII module in MMP-9 (Section 2.6.3, Figure 9). Initially, the SG moved towards the HPX domain and then remained located between the two domains, although closer to the HPX domain (Movie 5, Figure S14). A striking feature was that the N-terminal part of SG was also close to the catalytic domain, and the distance seemed stable from around 8 ns. ^134^R in the catalytic site formed an ionic bond with ^16^D in SG, and after approximately 14 ns, hydrogen bonds were formed between ^128^Y and ^160^ Y in the catalytic domain and ^37^S and ^34^P in SG. After around 17 ns, an additional ionic interaction was formed between ^25^E in SG with ^546^R (HPX, blade 1). This interaction was stable during the rest of the MD. After 27 ns, ^98^E and ^97^D in SG formed an interaction with ^685^R (HPX, blade 4). This interaction was more flexible with large distance variations.

Taken together, the MD simulations using the full-length proMMP-9 showed a structurally very flexible enzyme where the hinge region (OG domain) either was stretched out or coiled up, such that the two globular domains (catalytic and HPX domain) were either close to each other (closed conformation) or further from each other (stretched conformation). This was in accordance with experiments using SAXS along with AFM [29]. The MD simulations also indicated that SG could either be sandwiched between the FnII module and the HPX domain or tightly bound to one of these domains. Interactions observed during MD simulations reflected the interactions obtained by the peptide array experiments. Thus, the MD simulations along with the in vitro reconstitution experiments with the various deletion variants of proMMP-9 and the peptide arrays gave a picture of the complex nature of the interactions between the two macromolecules proMMP-9 and SG that resulted in the formation of the SDS-stable and reduction-sensitive proMMP-9·SG complex.

### 2.7. Conclusion

The in vitro reconstitution studies in the present work showed that when both the FnII module and the HPX domain were absent, the proMMP-9∙CSPG/SG complex was not formed, but in the presence of at least one of these two parts of proMMP-9, a complex was formed. This showed that even if the CSPG/SG core protein interacted with the amino acids that made up the pro-, catalytic- (minus the FnII part), and the OG-domain, the interaction was not sufficient to form the SDS-stable reduction-sensitive complex. This was also true with respect to the N- and O-linked glycans in the enzyme. The present work also showed that the CS chains in SG were not necessary for the complex formation.

Peptide arrays indicated that amino acids in the SG core protein located both at the N- and C-terminal to the central 18 amino acid Ser-Gly repeats were involved in the complex formation. Further, peptide arrays also showed that amino acids in the first and the second repeat in the FnII module were involved in the complex formation, as well as amino acids in the third and fourth blade of the HPX domain.

Docking and MD simulations suggested that the interactions between the SG core protein with the FnII module or HPX domain involved regions on each of the two interacting molecules that were located at considerable distances from each other. Therefore, individual amino acids that appeared important for binding in the 20-mer peptide arrays may not reflect that the amino acids were directly involved in the binding between the full-length molecules. When the SG core protein bound to the FnII module, the results suggested that at least a part of the SG core protein bound to the same or overlapping epitopes in the FnII module that bound gelatin. This fit well with our previous studies showing that gelatin inhibited the *in vitro* reconstitution of the proMMP-9∙CSPG/SG complexes and that the vast majority of the complexes isolated from THP-1 cells did not bind to gelatin-Sepharose [38,39]. The docking and MD simulations also suggested that the FnII module at the same time could interact with regions located both at the N- and C-terminal side of the Ser-Gly repeats in SG. One of the molecular models with the HPX domain also suggested this. MD simulations of the entire proMMP-9 also suggested that the hinge region (OG domain) was very flexible and that the two globular domains may be located close to or distant from each other. This observation was in agreement with previous results from SAXS and AFM [29]. The MD simulations also suggested that the SG core protein could be sandwiched between the FnII module and the HPX domain in the full-length enzyme due to the large flexibility of the long MMP-9 hinge region. Further, MD simulations indicated that amino acids in the catalytic domain of MMP-9 might be involved in the complex formation along with amino acids in the HPX domain and the FnII module. Overall, the peptide arrays along with docking and MD simulations indicated that no short single interacting linear motives in the two macromolecules could explain the strong SDS-stable and reduction-sensitive binding. In all, the results in the present work showed the complexity in the formation of the proMMP-9∙CSPG/SG complex(es).

## 3. Materials and Methods

### 3.1. Materials

Re-blot Plus Mild Solution (10x), TRIS, urea, guanidinium hydrochloride, DMSO, CaCl_2_·2H_2_O, citric acid, and sodium acetate were obtained from Merck (Darmstadt, Germany). 2-Methoxy-2,4-diphenyl-3(2H)-furanone (MDPF), (NH_4_)_2_SO_4_, and EDTA were from Fluka (Buchs, Switzerland). Acetic acid, acrylamide/bis-acrylamide, Coomassie Brilliant Blue G-250, and Triton X-100 were from BDH (Poole, UK). RPMI 1640, fetal bovine serum, streptomycin, penicillin, acrylamide/bis-acrylamide, sodium dodecyl sulfate (SDS; 20% in H_2_O), dithiothreitol (DTT), safranin O (No. S-2255), Ponceau S, cetylpyridinium chloride, sodium tetraborate, phorbol 12-myristate 13-acetate (PMA), HEPES, Brij-35, trypsin, soybean trypsin inhibitor (SBTI), silver nitrate, chondroitin sulfate C (shark cartilage CS), and gelatin (porcine skin, approximately 300 Bloom) were from Sigma (St. Louis, MO, USA). Proteinase-free chondroitin ABC lyase (cABC) and antibody against versican (2-B-1) were from Seikagaku Kogyo Co (Tokyo, Japan). Gelatin-Sepharose, heparin-Sepharose, Q-Sepharose, Sephadex G-50 (fine), Sephadex G-200, Sephacryl S-200, Sephacryl S-400, and protein G Sepharose were from GE-Healthcare (Uppsala, Sweden). Helix pomatia agglutinin (HPA) covalently linked to agarose beads was from EY Laboratories, Inc. (San Mateo, CA, USA). Unlabeled molecular weight standards were from BioRad (Richmond, CA, USA). Magic Marker molecular weight standards were from Invitrogen (Carlsbad, CA, USA). Western Blotting Luminol reagent was from Sancta Cruz (Santa Cruz, CA, USA). HRP-conjugated goat anti-rabbit secondary antibody was from Southern Biotech (Birmingham, AL, USA) and rabbit polyclonal antibodies against the C-terminal, mid-region, and N-terminal part of serglycin were from Antibodies-online Inc. (Aachen, Germany). Rabbit anti-rat MMP-9 polyclonal antibody (also detects mouse and human MMP-9) was obtained from Chemicon International Inc. (Temecula, CA, USA). The silver staining kit was from Pierce (Rockford, IL, USA). Peptide arrays and the soluble His-tag peptide were obtained from The Peptide Synthesis Core Facility, The Biotechnology Centre of Oslo, University of Oslo, Norway. Recombinant human his-tagged serglycin was from ProSpec (Ness Ziona, Israel). Vivaspin columns with a 10 and 30 kDa cut-off were from Satorius Stedim Biotech GmbH (Goettingen, Germany). Imperial blue protein stain, high range, and broad range molecular weight standards were from Thermo Scientific (Rockford, Il, USA). The recombinant catalytic domain of human MMP-9 containing the FnII repeats was from AnaSpec, Inc. (Fremont, CA, USA). The biotinylated protein ladder was from Cell Signalling (Danvers, MA, USA). Polyclonal rabbit anti-human MMP-9 carboxyterminal end (HPX domain) ab38906 was from Abcam (Cambridge, UK). The chromogenic substrate McaPLGLDpaAR-NH_2_ (ES001) was from R&D Systems, Inc (Minneapolis, MN, USA).

### 3.2. Biosynthesis of proMMP-9 and CSPGs

The human leukemic monocyte cell-line THP-1 was a kind gift from Dr. K. Nilsson, Department of Pathology, University of Uppsala, Sweden. The cells were cultured in RPMI 1640 medium with 10% fetal bovine serum, 50 μg/mL of streptomycin, and 100 units/mL of penicillin. To isolate secreted cell-synthesized CSPGs and proMMP-9, the cells were washed 3 times in serum-free medium and then cultured for 72 h in serum-free RPMI 1640 medium with or without 0.1 μM PMA, as described earlier [35]. Conditioned medium was harvested, and loose cells were pelleted by centrifugation at 1200 rpm (200g) for 10 min. ProMMP-9 and CSPGs were thereafter isolated and detected as described below.

### 3.3. Detection of PG-Bound CS Chains

PG-bound CS chains were quantified spectrophotometrically by the Safranin O color method as described previously [36].

### 3.4. Isolation of Secreted CSPG

Secreted CSPG from unstimulated THP-1 cells (monocytes) were isolated by Q-Sepharose anion-exchange chromatography as described previously [35,36,39]. Briefly, to the THP-1 conditioned medium was added urea and NaCl to a final concentration of 6 M and 0.35 M, respectively. This was applied to a Q-Sepharose column pre-equilibrated with 0.05 M of NaAc (pH 6.0) containing 6 M urea and 0.35 M NaCl (equilibration buffer). After a thorough wash with the equilibration buffer, the CSPG/SG was eluted from the column with an elution buffer (0.05 M NaAc containing 6 M urea and 1.5 M NaCl (pH 6.0)). The fractions containing CSPG/SG were pooled and diluted to give a NaCl concentration of 0.35 M, and urea was added to give a final concentration of 6 M. This material was re-subjected to another Q-Sepharose column pre-equilibrated with the equilibration buffer. After extensive washing with the equilibration buffer, the bound material was eluted with a gradient of NaCl (0.35–1.5 M) in 0.05 M NaAc, and 6 M urea (pH 6.0). The factions containing most CSPG/SG were pooled, desalted on Sephadex G-50 (fine) columns run in H_2_O, and concentrated in a Speed Vac (Savant). The amount of CSPG was based on the quantification of the GAG chains using the safranin O method and chondroitin sulfate C (shark cartilage CS) as a standard.

### 3.5. Purification of Serglycin

One milliliter of pooled CSPG (3 mg/mL) from Q-Sepharose anion-exchange chromatography was subjected to gel permeation chromatography on a Sephacryl S-400 column (90 × 1.6 cm) pre-equilibrated with 4 M guanidine hydrochloride and 50 mM sodium acetate, pH 6.0. The column was eluted with the same buffer, fractions collected, and PGs monitored by the safranin O method and characterized by SDS-PAGE and immunoblotting. Fractions with SG were pooled, diluted, and applied to a Q-Sepharose column. Eluted material from the Q-Sepharose column was desalted on a Sephadex G-50 column and thereafter concentrated on a vacuum centrifuge (Speed Vac). The amount of SG was determined by the safranin O method, using a standard curve generated from shark cartilage CS.

### 3.6. Chondroitin ABC Lyase Treatment

The PG bound CS chains were removed by digestion for 2 h at 37 °C with 0.2–1.0 units of cABC/mL in 0.05 M Tris-HCl, pH 8.0, containing 0.05 M sodium acetate [36].

### 3.7. Purification of proMMP-9 from the THP-1 Cells

The proMMP-9 in conditioned medium from PMA-stimulated THP-1 cells was partly purified as described previously [38,48]. Briefly, the THP-1 conditioned medium was first applied to a Q-Sepharose column to which CSPG/SG and proMMP-9∙CSPG/SG complexes bound, while proMMP-9 passed through the column. The proMMP-9 and proMMP-9 bound TIMP-1 were thereafter separated from TIMP-1 and other contaminants by applying the pass-through fraction to a gelatin-Sepharose column as described for the purification of the recombinant proMMP-9 deletion variants containing the FnII module (see Section 3.10). SDS-electrophoresis under reducing conditions, followed by either silver or Coomassie Blue staining, showed two bands, a major band at 92 kDa and a minor band at 28 kDa (Figure 2A). Western blotting revealed that the 92 kDa band was proMMP-9 (Figure 2C) and the 28 kDa band was TIMP-1 [38]. The amount of proMMP-9 was estimated spectrophotometrically at 280 nm using ε_280nm_ = 114.36 mM^−1^cm^−1^ [51], ignoring the contribution of TIMP-1.

ProMMP-9 was separated from TIMP-1 linked to its HPX domain on a Sephadex G-200 column in the presence of 0.1 % SDS. The equilibration and elution buffer of the Sephadex G-200 column contained 0.1 M HEPES pH 7.5, 0.15 M NaCl, 0.1% SDS, and 5.0 mM EDTA. The fractions containing pure 92 kDa proMMP-9 were concentrated on an Amicon Ultra Centrifugal Filter (Millipore) with a 10 kDa cut-off. This material was passed over a Sephadex G-50 column (equilibration and elution buffer 0.1 M HEPES pH 7.5 containing 5.0 mM EDTA) to remove SDS from the pure proMMP-9. This TIMP-1-free proMMP-9 was used to produce proMMP-9 polyclonal antibodies in rabbit (Eurogentec, Liège, Belgium). The obtained polyclonal antibodies did not react with other commercially obtained MMPs (MMP-1, MMP-2, and MMP-14), TIMP-1, or His-tagged serglycin.

### 3.8. Activation of proMMP-9

Activation of proMMP-9 from THP-1 cells was achieved by limited proteolysis with trypsin as described previously, and the activation was stopped by adding soybean trypsin inhibitor (SBTI) [37,48,62]. The enzyme activity of un-activated and trypsin-activated MMP-9 was determined with the fluorescence-quenched substrate Mca-PLGLDpaAR-NH_2_ (10 μM) in 0.1 M HEPES pH 7.5 (containing 10 mM CaCl_2_, 0.005% Brij-35) at 37 °C in a total volume of 100 μL. The initial rate of the reaction was determined on a Perkin Elmer LS 50 Luminescence spectrometer using the FL WinLab Software Package (Perkin Elmer). The reactions were followed for one minute, and during that time, 600 data points were collected. The excitation and emission wavelengths were λ_ex_ = 320 nm and λ_em_ = 405 nm and a slit width = 10 nm at both wavelengths.

### 3.9. Expression of Recombinant proMMP-9 Variants in Sf9 and High Five Insect Cells

The expression of recombinant full-length proMMP-9 (rpMMP-9) from Sf9 insect cells was performed as described previously [48]. Two deletion variants of MMP-9, one lacking the HPX domain (rpMMP-9ΔHPX) and the other lacking both the HPX- and the OG-domain (rpMMP-9ΔOGHPX) (Figure 1), were generated using the human preproMMP-9 cDNA (accession number: BC006093.1) cloned into the pReceiver-M02 vector (catalogue number: EX-F0125-M02, GeneCopoeia) as the template. A stop codon was inserted in the sequence of MMP-9 behind the codon for ^515^A or ^444^G, respectively. A twostep PCR reaction was used to produce rpMMP-9ΔHPX. In the first step, a stop codon was inserted before the coding sequence of the HPX domain using the primer pairs (fwd: 5′-TAG ACA TGA GCC TCT GGC AGC-3′, rev: 5′-GCT GGG TCT TAG GCA TCG TCC ACC GGA CTC AAA GG-3′). In the second step, the Invitrogen^TM^ Gateway^TM^ attB-sequences were added using the primer pairs (fwd: 5′-GGG GAC AAG TTT GTA CAA AAA AGC AGG CTT CGA AGG AGA TAG AAC CAT GAG CCT CTG GCA GC-3′, rev: 5′-GGG GAC CAC TTT GTA CAA GAA AGC TGG GTC TTA GGC ATC GTC CAC CGG ACT CAA AGG-3′). Finally, rpMMP-9ΔHPX was cloned into the pDONR221 plasmid using Gateway^®^ BP Clonase^®^ II Enzyme mix, according to Gateway^®^ protocol (Invitrogen, Thermo Fisher Scientific Inc.). To generate rpMMP-9ΔOGHPX, two STOP codons were introduced upstream of the coding sequence of the OG domain of preproMMP-9 using the primer pair (fwd: 5′-[Phos]TAA TAG CCT CGC CCT TAA CCT GAG CCA CG-3′, rev: 5′-[Phos]ACC ATA GAG GTG CCG GAT GCC A-3′), followed by a T4 ligation reaction. The template of the PCR reaction was preproMMP-9 previously cloned into the Gateway^®^ pDONR221 plasmid (pDONR-preproMMP-9) [48].

The deletion variant lacking the three FnII repeats in the catalytic site (rpMMP-9ΔFnII) (Figure 1) was generated by the use of phosphorothioate-modified primers, a technique previously described by Stoynova et al. [63]. In this variant, amino acids 216–390 were deleted from the full-length proMMP-9 by inverse PCR using phosphorothioate-modified primers (fwd: 5′-AAG GGC CAA GGA T*A*C* AGT TTG TTC CTC-3′, rev: 5′-TCC TTG GCC CTT G*C*C* CAG GGA CCA CAA CTC-3′). The nucleotides containing phosphorothioate were labelled with a star (*). The deletion variants rpMMP-9ΔFnIIHPX (lacked the FnII repeats and the HPX domains) and rpMMP-9ΔFnIIOGHPX (lacked the FnII repeats and both the HPX and the OG domain) (Figure 1) were cloned using the pDONR221 preproMMP-9ΔFnII constructs as a template for PCR. Two STOP codons were introduced behind the codon for ^515^A for generating rpMMP-9ΔFnIIHPX and behind the codon for ^444^G for generating rpMMP-9ΔFnIIOGHPX. The primer pairs used were fwd: 5′-[Phos]CTA TTA GGC ATC GTC CAC CGG ACT C-3′, rev: 5′-[Phos]TGA AAC GTG AAC ATC TTC GAC GC-3′ and fwd: 5′-CTA TTA TCA CTA ACC ATA GAG GTG CCG GAT GC-3′, rev: 5′-TAG TGA TAA TAG CCT CGC CCT GAA CCT GAG C-3′, respectively. The PCR reactions for rpMMP-9ΔFnIIHPX and rpMMP-9ΔFnIIOGHPX were followed by a T4 ligation reaction. The various pDONR-rpMMP-9 deletion constructs were subsequently cloned into BaculoDirect^TM^ Linear DNA (catalogue number: 12362013) using Gateway^®^ LR Clonase^®^ II Enzyme mix. Baculoviruses of all recombinant proMMP-9 variants were produced using Sf9 cells according to the protocol of the BaculoDirect^TM^ Baculovirus Expression System. The P3 and P4 viral stocks were used for the production of rpMMP-9ΔHPX or rpMMP-9ΔFnII in Sf9 cells in suspension and rpMMP-9ΔOGHPX, rpMMP-9ΔFnIIHPX, or rpMMP-9ΔFnIIOGHPX in High Five^TM^ cells according to the protocol (Invitrogen and Thermo Fisher Scientific).

### 3.10. Purification of Recombinant proMMP-9 Variants

The purification of recombinant proMMP-9 variants containing the FnII module in the catalytic site was performed as described previously [48]. Briefly, thirty milliliters of serum-free medium from Sf9 and High Five insect cells infected with baculovirus containing either rpMMP-9, rpMMP-9ΔHPX, or rpMMP-9ΔOGHPX were applied to a 1 mL column of gelatin-Sepharose pre-equilibrated with 0.1 M HEPES buffer pH 7.5 containing 5.0 mM CaCl_2_. After collecting the pass-through medium, the column was first washed with 10 column volumes of 0.1 M HEPES buffer pH 7.5 containing 5.0 mM CaCl_2_ and 1.2 M NaCl. This was followed by a new wash with 30–40 column volumes of 0.1 M HEPES buffer pH 7.5 containing 5.0 mM CaCl_2_. Bound proMMP-9 was eluted with a buffer containing 0.1 M HEPES pH 7.5, 5.0 mM CaCl_2_, and 7.5% DMSO. The eluted material was concentrated and depleted of DMSO (end DMSO less than 0.02%) using a spin column with a cut-off of 10 kDa. The amount of proMMP-9 in the sample was determined spectrophotometrically at 280 nm using the extinction coefficient ε_280nm_ = 114.36 mM^−1^cm^−1^ [51] for the full-length enzyme, ε_280nm_ = 73.185 mM^−1^cm^−1^ for the rpMMP-9ΔHPX, and ε_280nm_ = 73.060 mM^−1^cm^−1^ for the rpMMP-9ΔOGHPX (calculated from the ExPASy-ProtParam tool using the amino acid sequence). Previously, it was shown that determination of the protein concentration based on a theoretical calculation of a protein’s extinction coefficient at 280 nm from its predicted protein sequence from the DNA sequence was reliable [64]. Based on the ExPASy-ProtParam tool, an extinction coefficient of 112.73 mM^−1^cm^−1^ was obtained for the full-length proMMP-9, which was similar to the value of Murphy and Crabbe [51].

Purification scheme 1 of proMMP-9ΔFnII: Fifty milliliters of serum-free medium from Sf9 cells infected with baculovirus containing proMMP-9ΔFnII were first dialyzed twice against 2 L of 50 mM HEPES buffer with 10 mM CaCl_2_, pH 7.5. To this dialyzed material, 40 % (*w*/*v*) (NH_4_)_2_SO_2_ was added and mixed in a rotator for 20 min at 4 °C. Thereafter, the mixture was centrifuged at 15,000× *g* at 4 °C for 30 min. The supernatant contained proMMP-9ΔFnII, and more (NH_4_)_2_SO_2_ was added to the supernatant. This resulted in a salt concentration of 55% (*w*/*v*), and the sample was mixed in rotator for 20 min at 4 °C, followed by centrifugation as above. The precipitate containing proMMP-9ΔFnII was dissolved in 0.5 mL of gel filtration buffer (0.1 M HEPES buffer, 10 mM CaCl_2_, and 150 mM NaCl, pH 7.5). This material was applied to a Sephacryl-200 column (95 cm, diameter 1.2 cm) pre-equilibrated with gel filtration buffer. Fractions of 500 µL were collected, and the fractions containing proMMP-9ΔFnII (based on real-time gelatin zymography) were pooled and applied to a centrifugal filter with a 30 kDa cut-off. This resulted in removal of contaminating proteins with molecular sizes smaller than 30 kDa and a concentrated fraction of proMMP-9ΔFnII. The amount of proMMP-9ΔFnII was determined using ε_280nm_ = 79.550 mM^−1^cm^−1^ calculated from the ExPASy-ProtParam tool using the amino acid sequence.

Purification scheme 2 of proMMP-9ΔFnII: Fifty milliliters of serum-free medium from Sf9 cells infected with baculovirus containing proMMP-9ΔFnII were first desalted and concentrated on a spin column with a 30 kDa cut-off. Thereafter, twenty milliliters of this material were applied to a 3 mL column of heparin-Sepharose pre-equilibrated with 0.1 M HEPES buffer pH 7.5 containing 5 mM CaCl_2_. After first collecting the pass-through medium, the column was washed with 12 column volumes of equilibration buffer. The truncated enzyme was then eluted from the column with a 30 mL NaCl gradient (0–1.0 M) in the same buffer. The pooled eluted material was concentrated and depleted of NaCl using a spin column with a 10 kDa cut-off. The amount of proMMP-9ΔFnII was determined spectrophotometrically at 280 nm.

The purified and partly purified proMMP-9 variants were applied to gelatin zymography, real-time gelatin zymography, and SDS-PAGE (NuPAGE Novex 4–12% Bis-Tris gels). The SDS-PAGE gels were either further applied to Western blotting (using antibodies against MMP-9) or stained with Imperial blue where bands from the purified proMMP-9 variants containing the FnII module were cut out and sent to MS analysis at the Tromsø University Proteomics Platform (TUPP).

### 3.11. In Vitro Reconstitution of the proMMP-9·CSPG/SG Heteromer

*In vitro* reconstitution of proMMP-9∙CSPG/SG complexes was performed as described previously [39]. Briefly, purified proMMP-9 (0.05 µM) from THP-1 cells, ~0.05 μM trypsin activated proMMP-9, or 0.05 µM of full-length or truncated recombinant proMMP-9 were incubated with 230 µg/mL of purified CSPG/SG or 80 μg/mL of purified serglycin from untreated THP-1 cells. When rpMMP-9ΔFnII was used in the reconstitution experiments, a purity of approximately 50% was assumed. Hence, the amount of partly purified medium used was twice as much as expected from the A_280nm_ determination to give an enzyme concentration of approximately 0.05 μM. To ensure that sufficient amounts of rpMMP-9ΔFnIIHPX and rpMMP-9ΔFnIIOGHPX were used in the reconstitution experiments, different amounts of crude media containing these two variants and partly purified rpMMP-9ΔFnII were applied to real-time gelatin zymography. Based on this, the amount of crude media of rpMMP-9ΔFnIIHPX and rpMMP-9ΔFnIIOGHPX used in the reconstitution experiments contained approximately 3 to 5 times as much of these enzymes as used of the rpMMP-9ΔFnII deletion variant. These mixtures were incubated for 15 min to 24 h at 37 °C in 0.1 M HEPES buffer pH 7.5. In some experiments, these mixtures also contained 10 mM EDTA. After the incubation, the mixture was passed over a small Q-Sepharose column, washed with 10 column volumes of 0.05 M sodium acetate (pH 6.0) containing 6 M urea and 0.35 M NaCl, and then, the bound proMMP-9∙CSPG/SG complexes were eluted with the same buffer containing 1.5 M NaCl. Free full-length and deletion variants of proMMP-9 (not in complex with CSPG/SG) did not bind to the Q-Sepharose column under the above conditions. The fractions containing proMMP-9∙CSPG/SG complexes were pooled and desalted on Sephadex G-50 (fine) columns run in H_2_O. The volume was reduced in a Speed Vac (Savant). The purified CSPG/SG, SG, proMMP-9∙CSPG/SG, and proMMP-9∙SG complexes were then applied to gelatin zymography, real-time gelatin zymography, or Western blotting.

### 3.12. In Vitro Reconstitution of proMMP-9∙Serglycin Core Protein Complexes Lacking CS Chains

Either 100 μL of purified proMMP-9 from THP-1 cells (50 ng) or 100 μL Ht-SG (200 ng) in Western blotting buffer (48 mM TrisBase, 386 mM glycine, 20% methanol) were bound to a polyvinyl difluoride membrane using a slot blot apparatus. The different slots were cut out, and the membranes were blocked with milk powder as in Western blotting. Relevant membrane slots were thereafter incubated with either proMMP-9 (5 μg/mL) or Ht-SG (5 µg/mL) with or without 10 µg/mL His-tag peptide (Ht-P), washed and incubated with antibodies against proMMP-9, SG, or the His-tag (1:200). The membranes were thereafter washed and treated as described for Western blotting. Notice that the cut membrane slices contained the bound protein in the middle, surrounded by a membrane free of protein. Therefore, each membrane slice also contained its own control for unspecific binding.

### 3.13. Gelatin Zymography and Real-Time Gelatin Zymography

SDS-substrate PAGE was done as described previously [36] with gels (7.5 cm × 8.5 cm × 0.75 mm) containing 0.1% (*w*/*v*) gelatin in both the stacking and separating gel, 4 and 7.5% (*w*/*v*) of polyacrylamide, respectively. Gelatinase activity was evident as cleared (unstained) regions.

Real-time gelatin zymography was performed as described previously [48,65]. Briefly, zero--point-one percent (*w*/*v*) MDPF-fluorescent labelled gelatin was incorporated in the 7.5% SDS-PAGE separating gel, and 0.2% (*w*/*v*) MDPF-fluorescent labelled gelatin was incorporated in the 4.0% SDS-PAGE stacking gel. The fluorescent dye 2-methoxy-2,4-diphenyl-3(2H)-furanone (MDPF) was used to label gelatin to give MDPF-gelatin as described previously [65]. The main difference between normal gelatin zymography and real-time gelatin zymography is that in real-time zymography, the gel is not stained, and hence, it is possible to follow the degradation of the gelatin in real time without staining. Gelatinase activity was evident as dark bands against the un-degraded fluorescent background.

### 3.14. Western Immunoblotting Analysis

Purified proMMP-9 from THP-1 cells, recombinant full-length and deletion variants of human proMMP-9 from Sf9 and High Five insect cells, and cABC-treated purified SG from THP-1 cells with and without 0.1 M DTT were electrophoresed on SDS-polyacrylamide gel (NuPAGE Novex 4–12% Bis-Tris gels) and electroblotted to a polyvinyl difluoride membrane. After blockage of non-specific binding sites with non-fat milk in TBST (150 mM NaCl, 0.25% Tween-20, 20 mM Tris-HCL, pH 7.4), blots were incubated for 1 h at room temperature or 4 °C over night with either primary rabbit polyclonal antibody against human MMP-9 or rabbit polyclonal antibodies against SG (mixture of Ab against the C-terminal, N-terminal, and mid-region). After washing, the blots were incubated for 1 h at room temperature with an HRP-conjugated goat anti-rabbit secondary antibody. The blots were thereafter washed with TBST 3 × 5 min before visualization using Western Blotting Luminol reagent. The intensity of immunoblot bands was measured using a Luminescent Image Analyzer LAS-3000 with MultiGauge software Version 3.0 (Fujifilm, Tokyo, Japan).

Non-reduced samples of purified proMMP-9·CSPG complexes with the various proMMP-9 variants were applied to SDS-PAGE (4% acrylamide in the stacking gel and 7.5% acrylamide in the separating gel; identical concentrations as in gelatin-zymography). After electrophoresis, the polyacrylamide gels were incubated in 0.1 M DTT for 1 h at room temperature prior to blotting as described previously [36]. The gel was thereafter electro-blotted to a polyvinyl difluoride membrane, treated as described above to detect proMMP-9 variants bound to the CSPG/SG core protein. Reduced samples of purified proMMP-9·CSPG with the various proMMP-9 variants were applied to SDS-PAGE as describe above.

### 3.15. Peptide Arrays

Peptide arrays based on the amino acid sequences of SG (131 amino acids), the MMP-9FnII module (166 amino acids) and the MMP-9HPX domain (187 amino acids) were ordered from The Peptide Synthesis: Core Facility, The Biotechnology Centre of Oslo, University of Oslo. The first peptide in the peptide arrays was made up of the first twenty amino acids, and each successive peptide had a two amino acid shift. The membranes containing the peptide arrays of SG, the MMP-9FnII module, and the MMP-9HPX domain were first stained with 0.1% Ponceau stain in 5% HAc for 5 min at room temperature. These membranes were then dried, marked, and cut out as individual arrays of SG, the MMP-9FnII module, and the MMP-9HPX domain. The membranes were then fitted inside a 50 mL plastic tube, and the membrane was washed with water in a rotating wheel for 5 min. Then, the membranes were washed again with 10 mL TBST for 5 min after which the membranes were blocked with 10 mL blocking buffer (0.5 g non-fat milk powder in 10 mL TBST) for one hour in a rotating wheel to prevent nonspecific protein binding. Thereafter, the SG array membrane was incubated in 10 mL blocking buffer containing 8.8 µl of 11.2 µg/mL proMMP-9. The arrays of the MMP-9FnII module and the MMP-9HPX domain were incubated in 10 mL blocking buffer with 10 µL of 1 ng/μL Ht-SG. These incubations were done for 1–4 h at room temperature. The membranes were washed thereafter 3 times for 5 min with 10 mL TBST. Different primary antibodies according to the type of arrays were diluted in 10 mL blocking buffer and were added to the tubes with the arrays. In the tube with the SG array, the microliters of primary rabbit antibody against human MMP-9 (5.1 mg/mL) were added in 10 mL blocking buffer. Tubes with arrays of the MMP-9FnII module and the MMP-9HPX domain were incubated with 10 mL blocking buffer to which the following amounts of rabbit antibodies against human SG were added: 2 µL of C-terminal Ab (0.25 mg/mL), 8 µL of N-terminal Ab (1 mg/mL), and 8 µL of mid-region Ab (1 mg/mL). All membranes were incubated overnight on a rotating wheel at 4 °C. The next day, the membranes were washed with 10 mL TBST, 3 times 5 min, and then incubated with 10 mL blocking buffer containing 10 µL HRP-conjugated goat anti-rabbit secondary antibody for one hour at room temperature. Thereafter, the membranes were washed with 10 mL TBST, 3 times 5 min. Membranes were then developed in a 1:1 ratio of Luminol Reagent A and Luminol Reagent B (Western Blotting Luminol reagents) for antibody detection, and images were obtained by a luminescent image analyzer (Image quant LAS 4000 or Fujifilm LAS 3000).

To reuse membranes, they were stripped by first washing with 0.1M DTT in TBST for 1 h at 37 °C using a rotator. This was performed six times. Thereafter, the membranes were incubated in Re-blot Plus Mild Solution (1×) for 1 h at 37 °C using a rotator. To remove remnants of the DDT and Re-blot Plus Mild Solution, membranes were washed with TBST (5 min × 2). After this, membranes were ready to be reused.

### 3.16. Mutated Peptide Arrays

The amino acids selected for mutation were based on the results from the first peptide arrays. They were mutated as follows: The polar and charged amino acids serine (S), histidine (H), asparagine (N), glutamine (Q), tyrosine (Y), tryptophan (W), aspartate (D), glutamate (E), arginine (R), cysteine (C), and lysine (K) were mutated to alanine (A) since it has a small hydrophobic side chain. The polar amino acid threonine (T) was changed into valine (V). The hydrophobic amino acids valine (V), alanine (A), isoleucine (I), methionine (M), leucine (L), proline (P), and phenylalanine (F) were mutated to glycine (G), which lacks a side chain. Glycine (G) was mutated to leucine (L).

### 3.17. Construction of SG and proMMP-9 Models

PHYRE^2^ [66] was used to predict a structural model of SG. PHYRE^2^ is an online software tool, which predicts the structure based on multiple sequence alignments, secondary structure predictions, hidden Markov models of the query, and known 3D structures. After the predicted SG structure was acquired, the protein preparation wizard of the Maestro program was used to prepare the structure for docking.

A 3D model of proMMP-9 was built, using the Prime Homology Modelling program of the Schrödinger software package [67]. Briefly, the Fasta sequence of full-length proMMP-9 was downloaded and aligned with the X-ray structures of mini-MMP-9 containing the pro and catalytic domains with the FnII module (PDB id 1l6j; [31]) and HPX (PDB id 1itv; [30]). The Prime Homology Model program than predicted the structure of the hinge region (OG domain) between the catalytic and the HPX domains. The model consisting of the prodomain, the catalytic domain with the FnII module, the OG domain, and the HPX domain was then optimized using the Protein Preparation Wizard.

### 3.18. Docking

Docking of SG with FnII and HPX domains was performed with the protein-protein docking program BioLuminate of the Schrödinger software package [68]. The X-ray structures of FnII (PDB id 1l6j; [31]) and HPX (PDB id 1itv; [30]) were gathered from the PDB database (http://www.rcb.org/pdb/) and optimized using the protein preparation wizard tool of Maestro in the Schrodinger software package [67]. The structures were prepared by capping the N- and C-terminus, adding missing loop structures and hydrogen atoms. In addition, bond orders were assigned, and water molecules with less than 3 hydrogen bonds were deleted. The structure of HPX contains only the four blades, while the structure of FnII is comprised of the MMP-9 prodomain, catalytic region, and the three FnII repeats.

To validate the reliability and accuracy of the protein-protein docking program for the present molecular systems, chain B (HPX_B_) of the dimeric X-ray structure of HPX was docked with chain A (HPX_A_) from the same structure, using HPX_A_ as the fixed receptor. The docking was performed with standard mode, allowing maximum 100 docking poses. Protein interaction analysis was performed to analyze the amino acid interactions between the two HPX domains of the docked dimer and compare the interactions with the amino acid interactions in the X-ray structure (1itv) [30].

The optimized SG structure was docked to FnII and HPX, which were selected as receptors in the docking process. During the docking of SG with FnII, the maximum number of poses to return was set to 150, while the maximum number during the docking with HPX was 100. After docking, four steps were taken to select the best poses for further analysis. The different steps are given in Scheme 1.

In docking poses selected after the third step, five of the serine residues of the eight Ser-Gly repeats of SG were modified by adding the following 8 polysaccharide units, -Xyl-Gal-Gal-GlcA-GalNAc4S-GlcA-GalNAc4S-GlcA. The tetrasaccharide linker (Xyl-Gal-Gal-GlcA) was built using Maestro and linked to the tetrasaccharide CS repeat (GalNAc4S-GlcA-GalNAc4S-GlcA), extracted from the PDB Structure 1RWH [69]. After adding the chain of 8 polysaccharide units to SG, the selected docking poses were optimized in Maestro. The docking pose after optimization where the added polysaccharide chain did not hinder the interaction with FnII or HPX was selected for MD (Step 4 above).

### 3.19. Molecular Dynamics Simulations

Molecular dynamics simulations were performed with the docked HPX dimer, the X-ray structure dimer of HPX, two docking complexes of SG with FnII, four docking complexes of SG with HPX, the full-length proMMP-9 protein model, and four complexes of SG with the full-length proMMP-9 model. The program Desmond [67,70] was used for MD simulations. Simulations with the HPX dimer (docked and X-ray) were performed in order to test the accuracy of the structural predictions.

The simulations were performed for 100 ns, using the OPLS3e force field. However, the MD simulation with the full-length proMMP-9 model without SG was performed for 500 ns (Movie 1). All molecular systems were solvated with an orthorhombic box of simple point charge (SPC) water molecules, which were neutralized by adding Na^+^ and Cl^−^ ions. The width of the box was set to 10 Å. The NaCl concentration was 0.15 M. After this, the system setup was completed, and MD simulations were performed with default settings. Simulations were performed with the NPT ensemble (constant number of atoms (N), constant pressure (P): 1.01325 bar, constant temperature (T): 300 K). The time step was 2 fs, while the cut-off radius for non-bonded interaction was 9.0 Å. The calculation of non-bonded interactions was the most computationally demanding during MD simulations, and using a cut-off value for the long-range electrostatic and van der Waals interactions is therefore necessary when studying complex biological systems. In the present study, we used a non-bonded cut-off of 9 Å. Using a cut-off shorter than of 9 Å has been suggested to be likely to introduce simulation artifacts into electrostatic interactions, and 9 Å therefore represent a tradeoff between computational efficacy and accuracy [71]. After the simulation was finished, the “Simulation event analysis” command was used for analyzing the output from the three simulation trajectories.

## Figures and Tables

**Table 1:** Amino acid interactions between FnII and SG. Amino acid interactions between FnII and SG based on peptide arrays, docking, and MD simulations.

FnII				SG				Bond Type ^g^
Dock and MD Sim ^a^	Peptide Arrays			Dock and MD Sim ^a^	Peptide Arrays			
Amino Acid	Spots Contain Aa ^b^	Spots Bound to SG ^c^	Repeat Number ^d^	Amino Acid	Spots Contain Aa ^b^	Spots Bound to pMMP-9 ^e^	N- or C-Terminal ^f^	
G227(m)	1–2	-	1	N38(s)	10–19	14–15	N	Hb
D280(s)	19–28	19–22	1–2	R42(s)	12–21	14–15, 21	N	I
N282(s)	20–29	20–22	1–2	L120(s)	51–57	51–54	C	Hp
F294(s)	26–35	35	2	V96(s)	39–48	47–48	C	Hp
Q295 (s)	27–36	35	2	V96(s)	39–48	47–48	C	Hp
G306(m)	32–41	35,41	2	V96(m) E98(m)	39–49 40–48	47–48 47–48	C C	Hb Hb
R307(s)	33–42	35,41–42	2	S99(s)	41–50	47–48	C	Hb
S308(s)(m)	33–42	35,41–42	2	E98(s)	40–49	47–48	C	Hb
W313(s)	36–45	41–43,45	2	S99(s)	41–50	47–48	C	Hb
Y320(s)	39–48	41–43,45–48	2	V96(s)	39–48	47–48	C	Hp
K324(s)	41–50	41–43,45–49	2	F102(m)	42–51	47–48	C	Hb
F326(s)	42–51	41–43,45–49	2	S99(s) D100(s) A101(Cα)	41–50 41–50 42–51	47–48 47–48 47–48	C C C	Hp Hp Hp
F328(s)	43–52	41–43,45–49	2	S99(s) L120(s)	41–50 51–57	47–48 51–54	C C	Hp Hp

^a^ Amino acids based on the MD simulations of the best docked model of FnII with SG. ^b^ Amino acids belonging to the peptide spot in the peptide array of FnII and SG. ^c^ Peptide spots where the amino acids in FnII are represented after incubating the peptide arrays with SG. ^d^ To which FnII repeat the amino acid belongs. ^e^ Peptide spots where the amino acids in SG are represented after incubating the peptide arrays with proMMP-9. ^f^ The core protein of SG contains a Ser-Gly repeat zone from S67 to G84. The N-terminal represents the region before S67, and the C-terminal represents the region after G84. ^g^ The interaction type seen between the amino acid pairs between FnII and SG where I is ionic interaction, Hb is hydrogen bond, and Hp is hydrophobic interaction. (m): represents the main chain of the amino acid that is involved in the binding with another amino acid. (s): represents the side chain of the amino acid that is involved in the binding with another amino acid. (Cα): represents the Cα-atom of the amino acid that is involved in the binding with another amino acid. -: spots with no intensity, which could be due to no binding or too low signal/staining.

**Table 2:** Amino acid interactions between HPX and SG as seen in Model 1. Amino acid interactions between HPX and SG based on peptide arrays, docking, and MD simulations.

HPX				SG				Bond Type ^g^
Dock and MD Sim ^a^	Peptide Arrays			Dock and MD Sim ^a^	Peptide Arrays			
Amino Acids	Spots Contain Aa ^b^	Spots Bound to SG ^c^	Blade Number ^d^	Amino Acids	Spots Contain Aa ^b^	Spots Bound to pMMP-9 ^e^	N- or C-Terminal ^f^	
L659(s) ^1^(m) ^2^ L659(s)	61–70	69–70	3	L32(s) ^1^(m) ^2^ P34(s)	7–16 8–17	14,15 14,15	N N	Hp^1^, Hb^2^ Hp
D660(s) ^1^(m) ^2^ D660(s) D660(s)	61–70	69–70	3–4	L32(s) ^1^(m) ^2^ L33(m) S37(s)(m)	7–16 8–17 10–19	14,15 14,15 14,15	N N N	Hp^1^, Hb^2^ Hb Hb
Q675(s)	69–78	69–71, 76–78	4	L32(s)	7–16	14,15	N	Hp
D676(s)	69–78	69–71, 76–78	4	K39(s) P41(s) L43(s)	11–20 12–21 13–22	14,15 14,15,21 14,15,21	N N N	I Hp Hp
R677(s)	70–79	69–71, 76–78	4	L43(s)	13–22	14,15,21	N	Hp
W680(s)	71–80	69–71, 76–78	4	L32(s)	7–16	14,15	N	Hp
V691(s)	77–85	76–85	4	M29(s)	6–15	14,15	N	Hp
G695(Cα)	79–85	76–85	4	M29(s)	6–15	14,15	N	Hp
Y696(s) ^1,2^ Y696(s)	79–85	76–85	4	P28(s) ^1^(m) ^2^ L43(s)	5–14 13–22	14 14,15,21	N N	Hp^1^, Hb^2^ Hp
T698(s)	80–85	76–85	4	L43(s)	13–22	14,15,21	N	Hp
Y699(s) Y699(s) ^1,2^	81–85	76–85	4	D46(m) L47(s) ^1^(m) ^2^	14–23 15–24	14,15,21,23 15,21,23,24	N N	Hb Hp^1^,Hb^2^
D707(s)	85	76–85	4	L47(s)	15–24	14,15, 21,23–26	N	Hp

^a^ Amino acids based on MD simulations of one of the two best docked models of HPX with SG. ^b^ Amino acids belonging to the peptide spot in the peptide array of HPX and SG. ^c^ Peptide spots where the amino acids in HPX are represented after incubating the peptide arrays with SG. ^d^ To which HPX blade the amino acid belongs. ^e^ Peptide spots where the amino acids in SG are represented after incubating the peptide arrays with proMMP-9. ^f^ The core protein of SG contains a Ser-Gly repeat zone from S67 to G84. The N-terminal represents the region before S67, and the C-terminal represents the region after G84. ^g^ The interaction type seen between the amino acid pairs between HPX and SG where I is ionic interaction, Hb is hydrogen bond, and Hp is hydrophobic interaction. (m): represents the main chain of the amino acid that is involved in binding with another amino acid. The numbering (^1,2^) beside (m) correlates with the type of chain and the type of interaction on the binding amino acid in the table. (s): represents the side chain of the amino acid that is involved in the binding with another amino acid. The numbering (^1,2^) beside (s) correlates with the type of chain and the type of interaction on the binding amino acid in the table. (Cα): represents the Cα-atom of the amino acid that is involved in the binding with another amino acid.

**Table 3:** Amino acid interactions between HPX and SG as seen in Model 2. Amino acid interactions between HPX and SG based on peptide arrays, docking, and MD simulations.

HPX				SG				Bond Type ^g^
Dock and MD Sym ^a^	Peptide Arrays			Dock and Dyn Sym	Peptide Arrays			
Amino Acids	Spots Contain Aa ^b^	Spots Bound to SG ^c^	Blade Number ^d^	Amino Acids	Spots Contain Aa ^b^	Spots Bound to pMMP-9 ^e^	N- or C-Terminal ^f^	
K623(s)	43–52	-	3	D97(s) E98(s)	40–49 40–49	47,48 47,48	C C	I I
R634(s)	48–57	53–54, 56–57	3	E98(s)	40–49	47,48	C	I
R645(s)	54–63	54, 56–57	3	E98 (s)	40–49	47,48	C	I
S646(s)	54–63	54, 56–57	3	L113(s) P114(s)	48–57 48–57	47,48,50–54 47,48,50–54	C C	Hp Hp
A647(s)	55–64	56–57	3	L113(s)	48–57	47,48, 50–54	C	Hp
S648(s) ^1,2^	57–66	57	3	P114(s) ^1^ (m) ^2^	48–57	47,48, 50–54	C	Hp^1^ Hb^2^
D651(s) ^1^(m) ^2^	57–66	57	3	L43(s) ^1^ (m) ^2^	13–22	14,15, 21	N	Hp^1^ Hb^2^
R652(s) ^1^(m) ^2^ R652(s) ^1^(m) ^2^	57–66	57	3	S115(s) ^1,2^ Q118(s) ^1,2^	49–57 50–57	50–54 50–54	C C	Hp^1^,Hb^2^ Hp^1^,Hb^2^
M653(s)	58–67	-	3	P114(s) D116(s)	48–57 49–57	48,50–54 50–54	C C	Hp Hp
E687(s)	75–84	75–84	4	L120(s) S117(s)	49–57 50–57	50–54 50–54	C C	Hp Hp
L688(s)	75–85	75–85	4	S117(s) R42(s)	50–57 12–21	50–54 14–15,21	C N	Hp Hp
Q690(s)	76–85	76–85	4	I40(s)	11–20	14–15	N	Hp
V691(s)	77–85	77–85	4	I40(s)	11–20	14–15	N	Hp

^a^ Amino acids based on MD simulations of one of the best docked models of HPX with SG. ^b^ Amino acids belonging to the peptide spot in the peptide array of HPX and SG. ^c^ Peptide spots where the amino acids in HPX are represented after incubating the peptide arrays with SG. ^d^ To which HPX blade the amino acid belongs. ^e^ Peptide spots where the amino acids in SG are represented after incubating the peptide arrays with proMMP-9. ^f^ The core protein of SG contains a Ser-Gly repeat zone from S67 to G84. The N-terminal represents the region before S67, and the C-terminal represents the region after G84. ^g^ The interaction type seen between the amino acid pairs between HPX and SG where, I is ionic interaction, Hb is hydrogen bond, and Hp is hydrophobic interaction. (m): represents the main chain of the amino acid that is involved in binding with another amino acid. The numbering (^1,2^) beside (m) correlates with the type of chain and the type of bond on the binding amino acid in the table. (s): represents the side chain of the amino acid that is involved in the binding with another amino acid. The numbering (^1,2^) beside (s) correlates with the type of chain and the type of bond on the binding amino acid in the table. -: spots with no intensity, which could be due to no binding or not enough staining.

## Supplementary Materials

Supplementary materials can be found at https://zenodo.org/record/3905529#.XvLwcOcRXIV. Supplement 1: MD simulation of HPX dimer and docked HPX dimer. Supplement 2: MD simulation of the interactions between the SG core protein and the MMP-9 FnII module. Supplement 3: MD simulation of the interactions between the SG core protein and the MMP-9 HPX domain: Model 1. Supplement 4: MD simulation of the interactions between the SG core protein and the MMP-9 HPX domain: Model 2. Figure S1: Elution profile of Q-Sepharose purified CSPG from THP-1 cells using a Sephacryl S-400 column. Figure S2: Binding of pMMP-9 to a mutated SG peptide array. Figure S3: Binding of proMMP-9 to peptides in the array of SG. Figure S4: Binding of Ht-SG to a mutated FnII peptide array. Figure S5: Binding of Ht-SG to a mutated HPX peptide array. Figure S6: X-ray structure of the HPX dimer (PDB. 1itv), docking of HPX_B_ to HPX_A_, and MD simulations of both complexes. Figure S7: Docking and MD simulations of the interactions between the MMP-9FnII module and the SG core protein. Figure S8: Docking and MD simulations of the interactions between the MMP-9HPX domain and the SG core protein: Model 1. Figure S9: Docking and MD simulations of the interactions between the MMP-9HPX domain and the SG core protein:_Model 2. Figure S10: MD simulations of the full-length MMP-9. Figure S11: MD simulations of the interactions between the full-length MMP-9 and the SG core protein. Figure S12: MD simulations of the interactions between the full-length MMP-9 and the SG core protein. Figure S13: MD simulations of the interactions between the full-length MMP-9 and the SG core protein. Figure S14: MD simulations of the interactions between the full-length MMP-9 and the SG core protein based on the position from the best FnII-SG docking model. Movie 1, Movie 2, Movie 3, Movie 4, and Movie 5.

## Abbreviations

AFM	atomic force microscopy
APMA	p-amino-phenylmercuric acid
CS	chondroitin sulfate
CSPG	chondroitin sulfate PG
DTT	dithiothreitol
EDTA	ethylenediaminetetraacetate
FnII	fibronectin-II like
GAG	glycosaminoglycan
HPA	Helix pomatia agglutinin
HPX	hemopexin-like
HS	heparin/heparan sulfate
Ht-SG	His-tagged SG
MD	molecular dynamics
MMP	matrix metalloprotease
M9Ab	antibody against MMP-9
OG	O-glycosylated hinge domain
PG	proteoglycan
SAXS	small-angle X-ray scattering
SG	serglycin
SGAb	antibody against SG
TIMP	tissue inhibitor of metalloproteases
